# Altered neuronal physiology, development, and function associated with a common chromosome 15 duplication involving *CHRNA7*

**DOI:** 10.1186/s12915-021-01080-7

**Published:** 2021-07-28

**Authors:** Kesavan Meganathan, Ramachandran Prakasam, Dustin Baldridge, Paul Gontarz, Bo Zhang, Fumihiko Urano, Azad Bonni, Susan E. Maloney, Tychele N. Turner, James E. Huettner, John N. Constantino, Kristen L. Kroll

**Affiliations:** 1 Department of Developmental Biology, Washington University School of Medicine, 660 S. Euclid Avenue, Campus, Box 8103, St. Louis, MO 63110 USA; 2 Department of Pediatrics, Washington University School of Medicine, St. Louis, MO 63110 USA; 3 Department of Medicine, Division of Endocrinology, Washington University School of Medicine, St. Louis, MO 63110 USA; 4 Department of Neuroscience, Washington University School of Medicine, St. Louis, MO 63110 USA; 5 Department of Psychiatry, Washington University School of Medicine, St. Louis, MO 63110 USA; 6 Department of Genetics, Washington University School of Medicine, St. Louis, MO 63110 USA; 7 Department of Cell Biology and Physiology, Washington University School of Medicine, St. Louis, MO 63110 USA

**Keywords:** Induced pluripotent stem cells, Cortical neurons, Psychiatric disease, Neurodevelopmental disorders, Copy number variants, Chromosome 15q13.3 duplication, *CHRNA7*

## Abstract

**Background:**

Copy number variants (CNVs) linked to genes involved in nervous system development or function are often associated with neuropsychiatric disease. While CNVs involving deletions generally cause severe and highly penetrant patient phenotypes, CNVs leading to duplications tend instead to exhibit widely variable and less penetrant phenotypic expressivity among affected individuals. CNVs located on chromosome 15q13.3 affecting the alpha-7 nicotinic acetylcholine receptor subunit (CHRNA7) gene contribute to multiple neuropsychiatric disorders with highly variable penetrance. However, the basis of such differential penetrance remains uncharacterized. Here, we generated induced pluripotent stem cell (iPSC) models from first-degree relatives with a 15q13.3 duplication and analyzed their cellular phenotypes to uncover a basis for the dissimilar phenotypic expressivity.

**Results:**

The first-degree relatives studied included a boy with autism and emotional dysregulation (the affected proband-AP) and his clinically unaffected mother (UM), with comparison to unrelated control models lacking this duplication. Potential contributors to neuropsychiatric impairment were modeled in iPSC-derived cortical excitatory and inhibitory neurons. The AP-derived model uniquely exhibited disruptions of cellular physiology and neurodevelopment not observed in either the UM or unrelated controls. These included enhanced neural progenitor proliferation but impaired neuronal differentiation, maturation, and migration, and increased endoplasmic reticulum (ER) stress. Both the neuronal migration deficit and elevated ER stress could be selectively rescued by different pharmacologic agents. Neuronal gene expression was also dysregulated in the AP, including reduced expression of genes related to behavior, psychological disorders, neuritogenesis, neuronal migration, and Wnt, axonal guidance, and GABA receptor signaling. The UM model instead exhibited upregulated expression of genes in many of these same pathways, suggesting that molecular compensation could have contributed to the lack of neurodevelopmental phenotypes in this model. However, both AP- and UM-derived neurons exhibited shared alterations of neuronal function, including increased action potential firing and elevated cholinergic activity, consistent with increased homomeric CHRNA7 channel activity.

**Conclusions:**

These data define both diagnosis-associated cellular phenotypes and shared functional anomalies related to CHRNA7 duplication that may contribute to variable phenotypic penetrance in individuals with 15q13.3 duplication. The capacity for pharmacological agents to rescue some neurodevelopmental anomalies associated with diagnosis suggests avenues for intervention for carriers of this duplication and other CNVs that cause related disorders.

**Supplementary Information:**

The online version contains supplementary material available at 10.1186/s12915-021-01080-7.

## Background

Reciprocal copy number variants (CNVs) related to neurodevelopmental and neuropsychiatric disorders result from single-copy deletion or duplication of susceptible genomic intervals [1–3]. While CNVs involving deletion generally cause severe, highly penetrant patient phenotypes, CNVs involving duplication often exhibit widely variable and less penetrant phenotypic expressivity among affected individuals [4–6]. Among these, either chromosome 15q13.3 deletion or duplication causes clinical phenotypes including autism spectrum disorder (ASD), intellectual disability (ID), mood disorder, language delay, or schizophrenia [7–9]. 15q13.3 microdeletions usually cause severe cognitive deficits, behavioral abnormalities, and highly penetrant ASD [7, 10]. By comparison, 15q13.3 microduplication often causes milder phenotypes, including borderline ID, ASD, and attention deficit hyperactivity disorder (ADHD) [7, 11]. Notably, 15q13.3 microduplication is present in 1.25% of reported ADHD probands but also 0.61% of control subjects, suggesting that many individuals tolerate 15q13.3 duplication without a clinical diagnosis, due to its poor phenotypic penetrance [7, 8, 11].

15q13.3 microduplications either involve only the α-7 nicotinic acetylcholine receptor subunit gene (*CHRNA7*), with or without the first exon of *OTUD7A*, or instead duplicate multiple genes [7, 12–15]. *CHRNA7* is a prototypical genetic contributor to complex psychiatric disease: this protein family forms ligand-gated ion channels, which are stimulated by choline and acetylcholine to trigger calcium, sodium, and potassium cation flux [16]. *CHRNA7* duplication causes motor delays, hypotonia, ASD, ID, schizophrenia, and epilepsy, with particular phenotypes varying by individual [7, 15–17]. Although the clinical significance of increased CHRNA7 receptor dosage is unclear, no other copy number variation is detected in most reported cases, indicating that *CHRNA7* duplication is pathogenic, but with reduced and highly variable penetrance [7, 8, 16].

Patient-derived induced pluripotent stem cell (iPSC) models provide a powerful approach for modeling neurodevelopmental disorders. In recent years, we and others have characterized phenotypes associated with clinical diagnoses in these models, including modeling syndromic and de novo cases of ASD and monogenic or polygenic contributors to disease [18–22]. These studies identified diagnosis-associated phenotypes, including gene expression changes, differential regulation of developmental signaling, and altered neurogenesis and synaptogenesis [19–21, 23–26]. In addition to identifying these diagnosis-associated phenotypes, some studies found specific targets amenable to pharmacological rescue [18, 27, 28].

While CNVs at 15q13.3 involving *CHRNA7* duplication contribute to many neuropsychiatric disorders, consequences of these CNVs have not been extensively modeled. Mice with *CHRNA7* knockout, mimicking some 15q13.3 deletions, did not exhibit behavioral phenotypes [10, 11, 29]. *CHRNA7* duplication has not been modeled in vivo and the size of 15q13.3 duplications precludes their modeling by genome engineering in rodents. CHRNA7 overexpression in a mouse neuroblastoma cell line altered receptor sensitivity to choline and varenicline [30]. Two studies have characterized human iPSC-derived models involving duplications in the chromosome 15q region [28, 31]. One focused on a large duplication at 15q11-q13.1 involving 33 genes; a maternally expressed gene (*UBE3A*) was upregulated in this interval and this effect could be pharmacologically rescued [28]. A second study utilized three iPSC-derived excitatory neuron progenitor cell (NPC) models each from subjects with either 15q13.3 duplication or deletion [31]. These models exhibited increased or decreased CHRNA7 expression, respectively, while both duplication and deletion reduced CHRNA7-dependent calcium flux, indicating diminished channel activity. In the duplication models, diminished calcium flux was linked to elevated expression of several chaperone genes that control CHRNA7 protein folding and trafficking through the endoplasmic reticulum (ER) to the cell surface. Based upon these data, the authors proposed a model in which increased CHRNA7 levels impaired the efficiency of CHRNA7 trafficking through the ER, resulting in lower levels of CHRNA7 protein reaching the cell membrane, which diminished CHRNA7 channel activity [31].

*CHRNA7* duplications are common contributors to psychopathophysiology, exhibit widely variable phenotypic penetrance, and are difficult to model in animals. While there is some evidence for altered physiology of NPCs carrying *CHRNA7* duplications [31], potential contributors to the variable phenotypic penetrance among 15q13.3 duplication carriers are entirely unknown. How this CNV more broadly affects neurodevelopment, global gene expression, and electrophysiological function of neurons has also not been evaluated. Therefore, here we generated iPSC models from two first-degree relatives with the same 15q13.3 copy number variant, which duplicates only *CHRNA7*. These subjects include a boy with distinct features of autism and emotional dysregulation (the affected proband, AP) and his clinically unaffected mother (the UM). These models were compared to unrelated male and female control subjects lacking this duplication (the UC-M and UC-F). We used these models to assess the consequences of *CHRNA7* duplication on the development and function of both cortical excitatory neurons (cExN) and inhibitory interneurons (cIN), as disruption of both neuronal cell types frequently contributes to neurodevelopmental disorders. This work defined a suite of phenotypic penetrance-associated neurodevelopmental anomalies in the AP, including deficits in neurite extension, neuronal migration, neuronal maturation, and ER stress, and related perturbations of neuronal gene expression. While these neurodevelopmental deficits were not present in the other model with *CHRNA7* duplication (UM), both models carrying the duplication (UM and AP) shared multiple functional abnormalities, as defined by electrophysiology, including increased action potential firing and enhanced choline responsiveness.

To our knowledge, this is the first cellular modeling study to characterize cases of *CHRNA7* duplication involving two first-degree relatives with differential diagnoses. A range of molecular, cellular, and functional assays was used to define alterations of neuronal gene expression, neurodevelopment, and altered functional properties; these were linked either to phenotypic penetrance or to *CHRNA7* duplication, both with and without diagnosis, and some diagnosis-associated phenotypes were amenable to pharmacological rescue. The distinct neuronal anomalies related to clinical phenotype versus *CHRNA7* duplication that were defined here could contribute to the differential phenotypic penetrance seen in other duplication carriers and provide models for pre-clinical testing of potential interventions for individuals with this disorder.

## Results

### iPSC-derived neural progenitor cells from the affected proband exhibit increased proliferation

The selected pedigree of four individuals includes three subjects with the same 15q13.3 duplication, the mother, who has no clinical diagnosis (UM), her older son, who exhibits distinct features of autism and emotional dysregulation (the affected proband, AP), and his younger, affected brother, who exhibits mild ASD, ADHD, and mood disorder traits. The family pedigree and the clinical phenotypes of these three mutation carriers are summarized in Table 1 and Additional file 1. Chromosomal Microarray Analysis (CMA) defined one CNV in these three subjects, a ~400 kilobase gain at chromosome 15, band q13.3 (see the “Methods” section, Table 1, Additional file 1). Only one gene, *CHRNA7*, is in the duplicated region. The father in this pedigree does not carry this duplication (Additional file 1).

**Table 1:** Clinical phenotypes of individuals in the pedigree under study

	Unaffected mother (UM)	Affected proband (AP)	Affected brother
Age at first assessment	N/A	8 years old	N/A
Sex	Female	Male	Male
Social Responsiveness Scale-2 by mother	N/A	72	N/A
Child Behavioral Checklist (CBCL) total by mother	N/A	72	N/A
Attention deficit (AD) by CBCL	N/A	92	N/A
Screen for Child Anxiety-Related Disorders (SCARED)-P score by mother	N/A	46	N/A
Pervasive Developmental Disorder (PDD)	No	Yes	Yes
Depression and anxiety disorder (by Teacher Report Form-TRF)	No (some traits self-reported)	Yes Score-71	Yes
Seizure history	No	No	No
Developmental delay	No	Yes-Language development	Yes
Eye contact	Normal	No eye contact at the age of five, intermittent at the age of 12	Normal
Nonverbal communication problems	No	Yes	Yes
Speech/Language Delay	No	Yes	Yes
ADHD	No (some traits self-reported)	Yes	Yes
ASD	No	Yes (level 1)	Yes (traits)
Mood disorder	No	Yes	Yes
Genetic variant	424 kb gain at 15q13.3	424 kb gain at 15q13.3	444 kb gain at 15q13.3
hg19 coordinates of variant	[32,020,432-32,444,044]	[32,019,918-32,444,044]	[31,999,631-32,444,044]

Clinical phenotypes and location of copy number variation in a family with 15q13.3 duplication, including the unaffected mother (UM), affected proband (AP), and his affected brother. Note that the minor differences in reported lengths of the duplications between subjects reflect the limits of resolution of detection by chromosomal microarray analysis

**Fig. 1:**
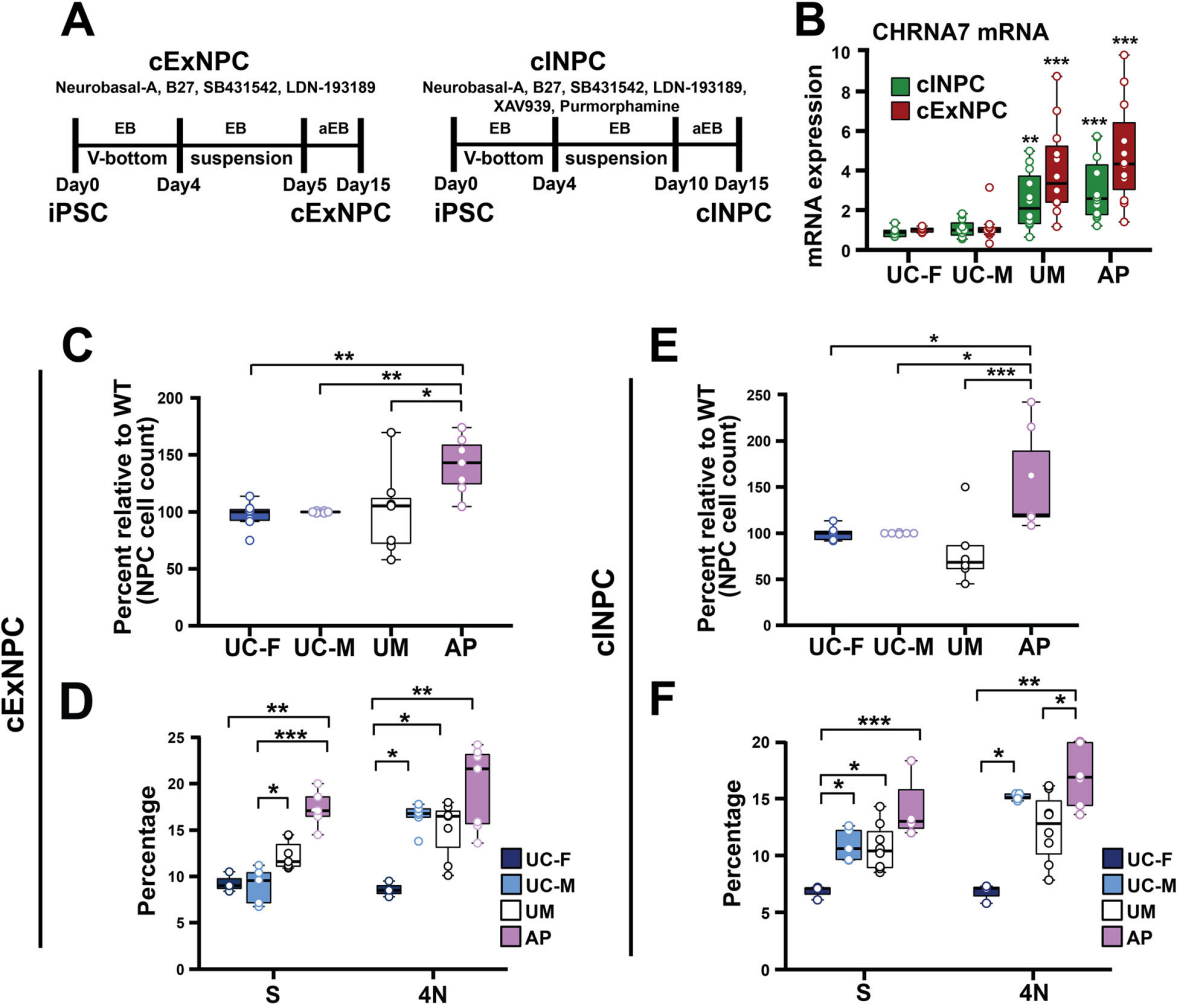
Characterization of cExNPCs and cINPCs from patient-derived iPSCs. **a** Differentiation schemes for generating cExNPCs and cINPCs, including timeline, small molecules used, and maintenance as a suspended or adherent embryoid body (EB/aEB). **b** CHRNA7 expression in cExNPCs and cINPCs was analyzed by RT-qPCR. Data is the average +/-SEM from four independent biological replicate experiments (*n* = 4). **c** cExNPCs were seeded in equal numbers for each line tested and counted after four days of maintenance (*n* = 7 biological replicates). **d** cExNPCs were stained with propium iodide for DNA content and analyzed by FACS. S and 4N (M phase) cells were quantified for each study subject. Average values are shown from seven independent biological replicates (*n* = 7). **e** cINPCs were seeded in equal numbers for each line tested and counted after 4 days of maintenance (*n* = 7 biological replicates). **f** cINPCs were stained with propidium iodide for DNA content and analyzed by FACS. S and 4N (M phase) cells were quantified for each model. Values are from seven independent biological replicate experiments (*n* = 7). All significant findings in this manuscript were confirmed in three or more independent biological replicate experiments performed using two clonal lines for the UM and AP models, and one clonal line for the UC-F and UC-M models, as summarized in Additional file 4. Clones used, replicates, and data values are in Additional file 4. Plot shows the median value, calculated by using a Kruskal-Wallis non-parametric test as described in the Methods. *P* values: *P < 0.05, **P < 0.01, and ***P < 0.001

Renal epithelial cells from the UM and AP were reprogrammed to generate three clonal iPSC lines per subject and compared with single clonal iPSC lines derived from unrelated, unaffected male and female control subjects (UC-M and UC-F). All lines exhibited similar morphology, expressed the pluripotency marker POU5F1, and were karyotypically normal (Additional file 2). The development and/or function of cortical excitatory neurons (cExNs) and inhibitory cortical interneurons (cINs) is frequently disrupted to contribute to neurodevelopmental disorders [32, 33]. Therefore, we used two clonal lines each from the UM and AP, and one clonal line each from the UC-M and UC-F, to generate cExN and cIN neural progenitor cells (cExNPCs and cINPCs) (Fig. 1a). After 15 days of NPC specification, cExNPCs and cINPCs were maintained as a monolayer. CHRNA7 expression levels, assessed by RT-qPCR, were significantly increased in both AP- and UM-derived NPCs, relative to UC-M or UC-F-derived NPCs (Fig. 1b). During cExNPC and cINPC maintenance, AP-derived NPCs exhibited more rapid proliferation than UM, UC-M, or UC-F-derived NPCs, as measured by seeding equal cell numbers and quantitation after four days (Fig. 1c, e). We further assessed cell cycle kinetics by FACS analysis of propidium iodide (PI)-stained cExNPCs and cINPCs, finding that AP-derived NPCs had significantly higher percentages of both S- and M-phase cells than one or more of the unrelated control NPCs. Percentages of cells in S and 4N phases of the cell cycle are shown for cExNPCs and cINPCs, respectively (Fig. 1d, f), with summary data for all cell cycle phases in Additional file 3, and biological replicate experiments and clones used for each experiment in this manuscript summarized in Additional file 4.

### Defects in neurite extension and production of VGAT-expressing punctae in AP-derived neurons

**Fig. 2:**
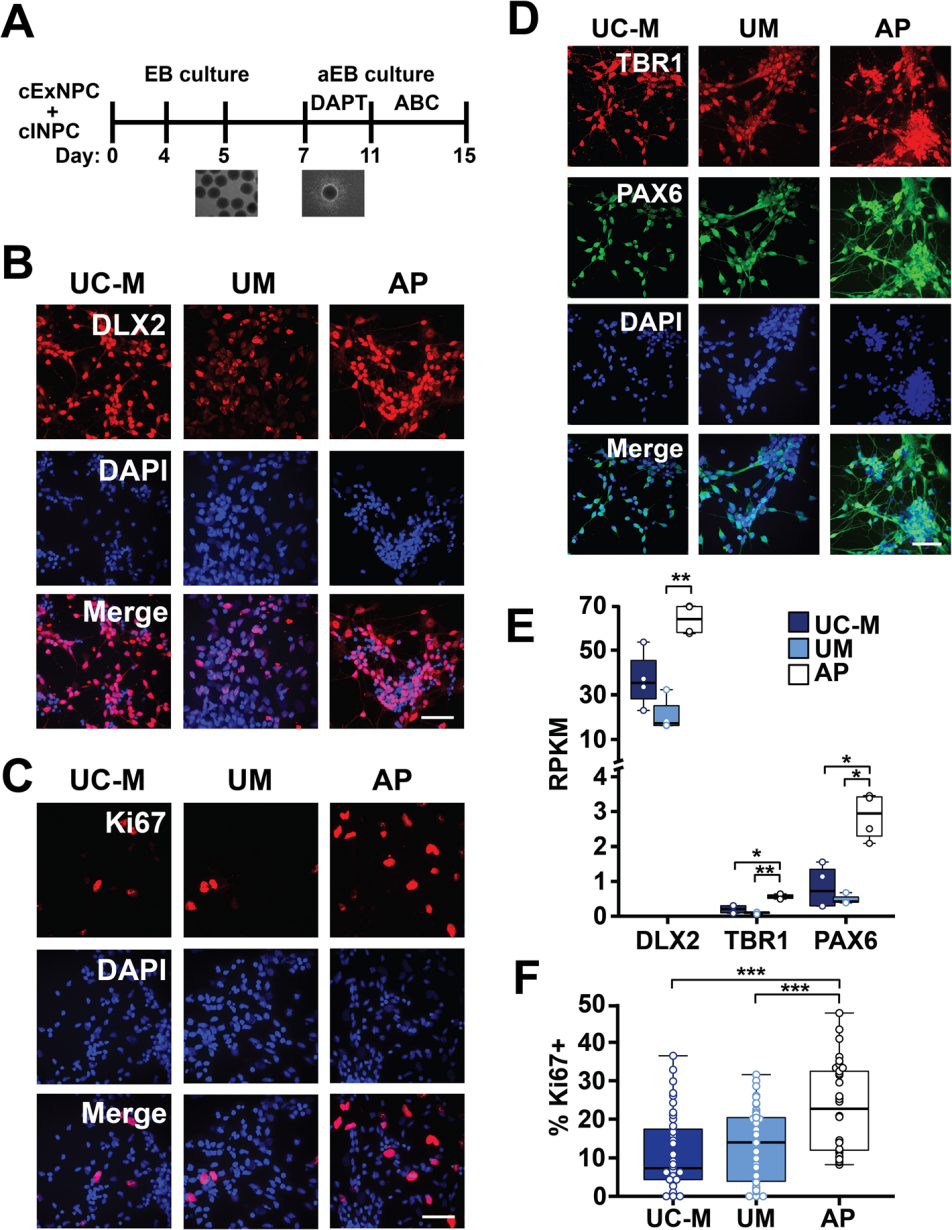
Generation and characterization of cortical neuroids. **a** Schematic of method used to generate cortical neuroids, by combining cExNPCs and cINPCs (1:1 ratio) and differentiating and maturing them for 15 days. ABC=ascorbic acid, BDNF, and cAMP. See the “Methods” section for further details. **b**–**d** Immunocytochemistry with antibodies indicated detects cINPCs (DLX2), proliferating NPCs (Ki67), and cExNPCs (TBR1 and PAX6), with representative images from one clonal line per subject shown. **e** RNA-seq analysis defined differences in gene expression between the AP, UM, and UC-M neuroids for the markers shown (*n* = 4 independent biological replicates from one clonal line per subject). **f** Immunocytochemical quantification of the percentage of cells expressing the proliferative marker Ki67, *n* = 4 biological replicate experiments utilizing two clonal lines for the AP and UM and one clonal line for the UC-M. Clones used, replicates, and data values are in Additional file 4. Scale bars=50μm

**Fig. 3:**
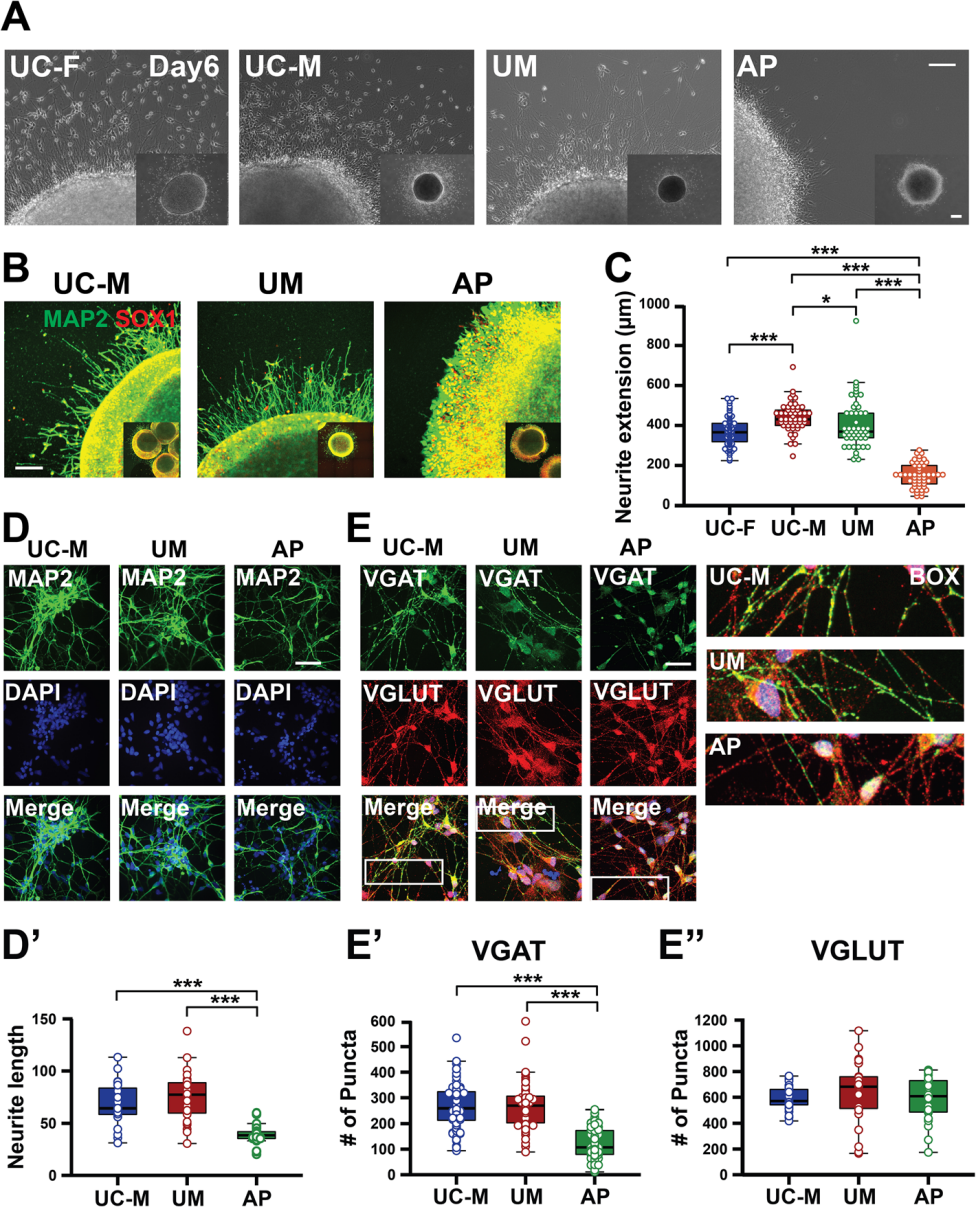
Morphometric analysis of differentiated cortical neuroids. **a**–**c** Five days after plating cortical neuroids in differentiation media, neurite length was analyzed for each sample type. **a** Representative light microscopy images are shown. Quantification was performed as described in the “Methods” section, by defining the distance between the border of the plated neuroid and the tips of neurites extending from that neuroid on a per-EB basis. Scale bar=250 μm. **b** Immunocytochemical analysis of neurite extension using MAP2 staining, with representative images shown. Scale bar=150 μm. **c** Quantification of neurite extension is shown for seven biological replicate experiments (*n* = 7). Plot shows the median value, calculated by using Kruskal-Wallis non-parametric tests, as described in the Methods. **(d-d’)** Neurite length was analyzed in plated MAP2 immunostained neurons, using data from three independent biological replicate experiments (*n* = 3). **(e-e”)** Expression of the GABA and glutamate transporters, VGAT and VGLUT, was assessed by immunocytochemical analysis of these neurons. Representative images are shown in **e** and synaptic puntae were quantified in (**e’**-**e”**) for VGAT (**e’**) and VGLUT (**e”**). Data were derived by quantifying ~15 stained neuroids derived from four independent biological replicate experiments (*n* = 4). Clones used, replicates, and data values are in Additional file 4. Scale bar=75 μm. Plot shows the median value, calculated by using Kruskal-Wallis non-parametric tests as described in the “Methods” section. *P* values: *P < 0.05, **P < 0.01, and ***P < 0.001

To assess the differentiation potential of the UC-M, UM, and AP lines, cExNPCs and cINPCs were combined at a 1:1 ratio to generate cortical neuroids (Fig. 2a). This co-culture accelerates neuronal differentiation and maturation and models interactions between cortical excitatory and inhibitory neurons that occur during cortical development in vivo. Upon dissociation and plating of these day 15 neuroids, AP-derived neuroids exhibited increased expression of NPC markers DLX2, TBR1, and PAX6, versus UM and UC neuroids, and also had higher frequencies of cells expressing the proliferative marker Ki67 (Fig. 2b–f). This could relate to increased AP-derived NPC proliferation (Fig. 1). We also plated differentiating neuroids without dissociation, allowing them to extend neurites. By 5 days, AP-derived neuroids exhibited a neurite extension deficit not seen in either UM- or UC-derived neuroids. This was quantified using light microscopy and further visualized by MAP2 staining (Fig. 3a–c). On differentiation day 15, neuroids were also dissociated and plated neurons assessed by immunocytochemistry (ICC) for MAP2, confirming neurite length deficits in AP-derived neurons (Fig. 3d–d’). ICC for GABA and glutamate transporters, VGAT and VGLUT, further assessed synaptic vesicle formation and transport in cINs and cExNs, respectively. While VGLUT-expressing punctae were present in cExN neurites derived from all three lines, AP-derived cINs exhibited significantly reduced formation of VGAT-expressing punctae along neurites, versus UM and UC-M models (Fig. 3e–e”). Both increased expression of NPC and proliferative markers and impaired acquisition of characteristics of differentiated and mature neurons suggested that the AP-derived neurons were relatively immature, relative to UM-, UC-F-, and UC-M-derived neurons.

### Transcriptome comparisons in cortical neuroids

**Fig. 4:**
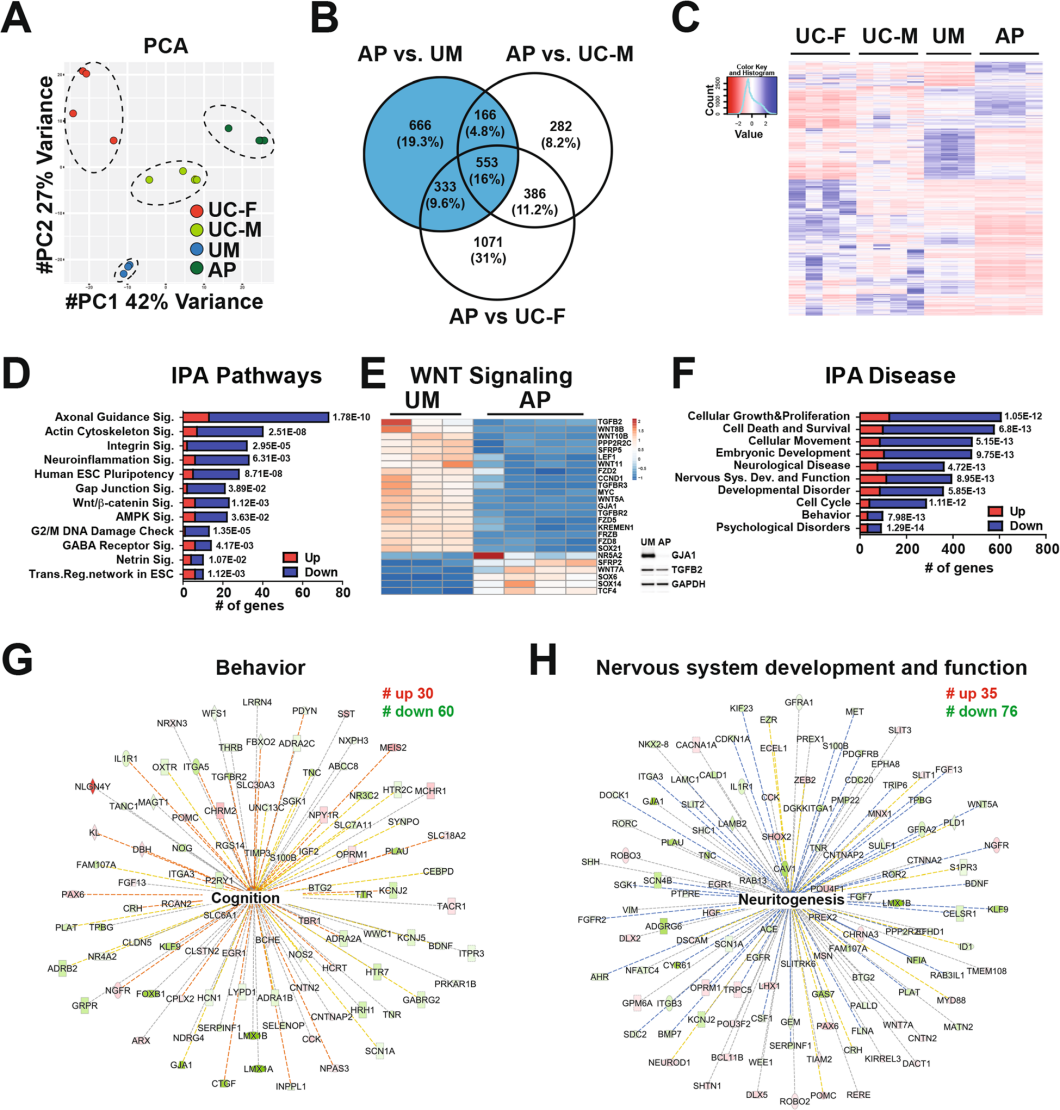
Transcriptomic analysis of differentiated cortical neuroids, defining differential gene expression between the AP and UM. RNA-seq was conducted on cortical neuroids after 15 days of differentiation. **a** Principal Component Analysis (PCA) of gene expression in differentiated neuroids derived from the UC-M, UC-F, UM, and AP models is displayed as a multidimensional scaling plot derived from four independent biological replicate experiments (*n* = 4) performed using one clonal line for each subject. Data values are in Additional file 5. **b** Venn diagram showing differentially expressed genes (DEGs) defined by pairwise comparisons of the AP versus (vs.) UM, AP vs. UC-M, and AP vs. UC-F datasets. AP vs. UM-specific DEGs are shown in blue. **c** These AP-specific DEGs were further analyzed by hierarchical clustering analysis, with the other samples included for comparison. **d**–**h** DEGs were assessed by Ingenuity Pathway Analysis (IPA), identifying **d**–**e** AP-enriched pathways, and **f** disease-related GO terms. **d** IPA-pathway analysis identified differential enrichment for Wnt signaling-related gene expression, with expression of these genes visualized as a heat map in **e**; inset at right shows protein levels of two targets in the UM and AP, as assessed by Western blotting, with GAPDH as a loading control. In **d** and **f**, the number of genes related to each term is represented on the x-axis, while red and blue color indicates up and downregulated genes, respectively. *P* values for each term are indicated to the right of each bar. **f** IPA disease GO terms identified gene networks associated with **g** behavior and **h** nervous system development and function. Numbers (#) of upregulated and downregulated genes in each network are indicated. Within each network, red and green symbols indicate upregulated and downregulated genes, respectively, while color intensity indicates the relative degree of differential expression

To identify gene expression differences distinguishing neurons from the four models, we performed RNA-seq analysis on cortical neuroids after 15 days of differentiation. Four independent biological replicates were generated and clustered by Principal Component Analysis (PCA) of processed reads (Fig. 4a). We defined differentially expressed genes (DEGs) by pairwise comparisons between sample types (FDR <0.05 and log2 fold-difference >1 cut-off values; see Methods, Additional file 5). We focused first on genes that were differentially expressed in the AP versus (vs.) the UM, as these subjects have a shared genetic background including *CHRNA7* duplication, but exhibit differential phenotypes (Fig. 4b, blue). Hierarchical clustering of these DEGs, with the UC samples included for comparison, demonstrated that most exhibited increased expression in the AP, versus the UM (Fig. 4c). We used Ingenuity Pathway Analysis (IPA) to define enriched pathways and disease-related networks for AP-specific DEGs (Additional file 6). These included molecular pathways involved in axon guidance signaling, the cell cycle, Wnt signaling, GABA receptor activity, neuroinflammation, and gap junction signaling (Fig. 4d; Additional file 6), with most Wnt signaling-related genes exhibiting diminished expression in AP- versus UM-derived neurons (Fig. 4e). IPA disease network analysis also defined clusters of DEGs related to nervous system development, developmental disorders, behavior, and psychological disorders (Fig. 4f). For example, genes related to cognition and neuritogenesis exhibited predominantly reduced expression in AP-derived neurons (Fig. 4g-h). This was congruent with AP-specific neurite extension deficits (Fig. 3a).

We also curated AP-specific DEGs by comparison with both UM-, UC-M-, and UC-F-derived neurons (1052 genes; Additional file 7A). These included many genes with diminished expression in the AP relative to all other models (Additional file 7B) and encompassed many molecular pathways and disease-related GO terms similar to those identified in AP versus UM comparisons, including Wnt, axon guidance, and GABA receptor signaling, nervous system development, and psychological disorders (Additional file 7C-F; Additional file 8). For example, gene clusters related to neurite growth and neuronal migration had predominantly reduced expression in the AP versus other models (Additional file 7C-F; Additional file 8), suggesting that similar pathways exhibited impaired expression in the AP, by comparison with neurons from both related (UM) and unrelated (UC) models.

As another way to identify specific pathways that could be contributing to the AP's differential affectation, we cross-referenced all genes with differential expression between the AP and the three other models (UM, UC-M, UC-F) with genes that have genome-wide significance for de novo protein-coding variants associated with neurodevelopmental disorders [34, 35] or that were significant in a transcription-wide association study (TWAS) of autism [36]. The intersecting genes included *ANXA1*, *BCL11A*, *BCL11B*, *CACNA1A*, *COL2A1*, *DSCAM*, *ERF*, *FBN1*, *FOXP2*, *GATA3*, *KCNJ6*, *KCNS3*, *KIF11*, *MEIS2*, *NFIA*, *PRKAR1B*, *SATB1*, *SLC6A1*, *SPRY2*, *TBR1*, *TCF4*, *TMEM42*, *TRPM3*, and *ZEB2* (Additional file 8). To look at potential genetic interactions, physical interactions, co-expression, shared protein domains, and co-localization we entered this list into GeneMania [37], which defined a network of dysregulated DEGs (Additional file 7G). We also tested whether there was specifically an enrichment of protein-protein interactions (PPI) using STRING-DB [38]. In this PPI analysis, we observed 15 edges and expected 4 edges which showed an enrichment (*p* = 6.62 x 10^-6^). Gene ontology analysis of this network revealed enrichments for molecular functions including DNA-binding transcription repressor activity (FDR = 1.0 x 10^-5^) and voltage-gated cation channel activity (FDR = 6.9 x 10^-3^), suggesting that the genes in the categories that are shown in Additional file 7G may be contributors to the AP’s clinical phenotype.

### UM-specific differential gene expression in cortical neuroids compared to unrelated controls

Since both related models (AP/UM) carry the same *CHRNA7* duplication, we also defined UM-specific DEGs, by comparison with unrelated controls (Additional file 9A). A DEG cluster exhibited increased expression, versus all other samples, while UM- and AP-derived neurons also shared a cluster with decreased expression versus the UC samples (Additional file 9B). UM-specific DEGs exhibited enrichment for some of the same gene ontology (GO) terms identified for AP-specific DEGs (e.g., GABA receptor and gap junction signaling, behavior/psychological disorders; Additional file 9C-D; Additional file 10). However, many DEGs and enriched GO terms and pathways obtained from UC model comparisons differed between the UM and AP. For example, Wnt signaling-related genes were not differentially expressed in the UM versus UC comparisons. Furthermore, while UM-specific DEGs also included a neuritogenesis-related network, genes in this network were more highly expressed in the UM versus the UC-M and UC-F models (Additional file 9E-F), while neuritogenesis-related genes had diminished expression in the AP, versus both UM and UC models (Fig. 4h, Additional file 7F). These DEGs included cytoskeletal genes involved in neuritogenesis, including ACTG2, ACTN1, and/or ACTN2 [39–43]; these genes exhibited diminished expression in the AP versus the UM and UC controls, but elevated expression in the UM relative to the unrelated controls. We used cortical neuroids derived from a second clonal line for the AP and UM models to confirm a subset of these findings by RT-qPCR, obtaining findings congruent with the RNA-seq analysis (Additional file 11A-B).

To assess the relative contributions that sex, age, or the individual genetic backgrounds of the subjects used to derive these models may have had on the identification of DEGs in this work, we also performed variancePartition analysis. This analysis indicated that the individual from whom the samples were derived was the major contributor to differential expression, while the age and sex of study subjects were minor contributors (Additional file 11C). We further characterized the contribution that sex of the model may have had on the identification of DEGs by comparing each set of DEGs (Additional file 5) with genes that were observed to be sex-differentially expressed in the human brain in a recent study [44], finding that fewer than 1% of the DEGs identified here were previously characterized as exhibiting sex-differential expression (Additional file 5, right columns). This is congruent with the covariance analysis and suggests that the sex of the samples was not a major contributor to the DEGs identified.

### Interneuron migration is diminished in AP-derived neurons and this defect is partially reversed by a Wnt agonist

**Fig. 5:**
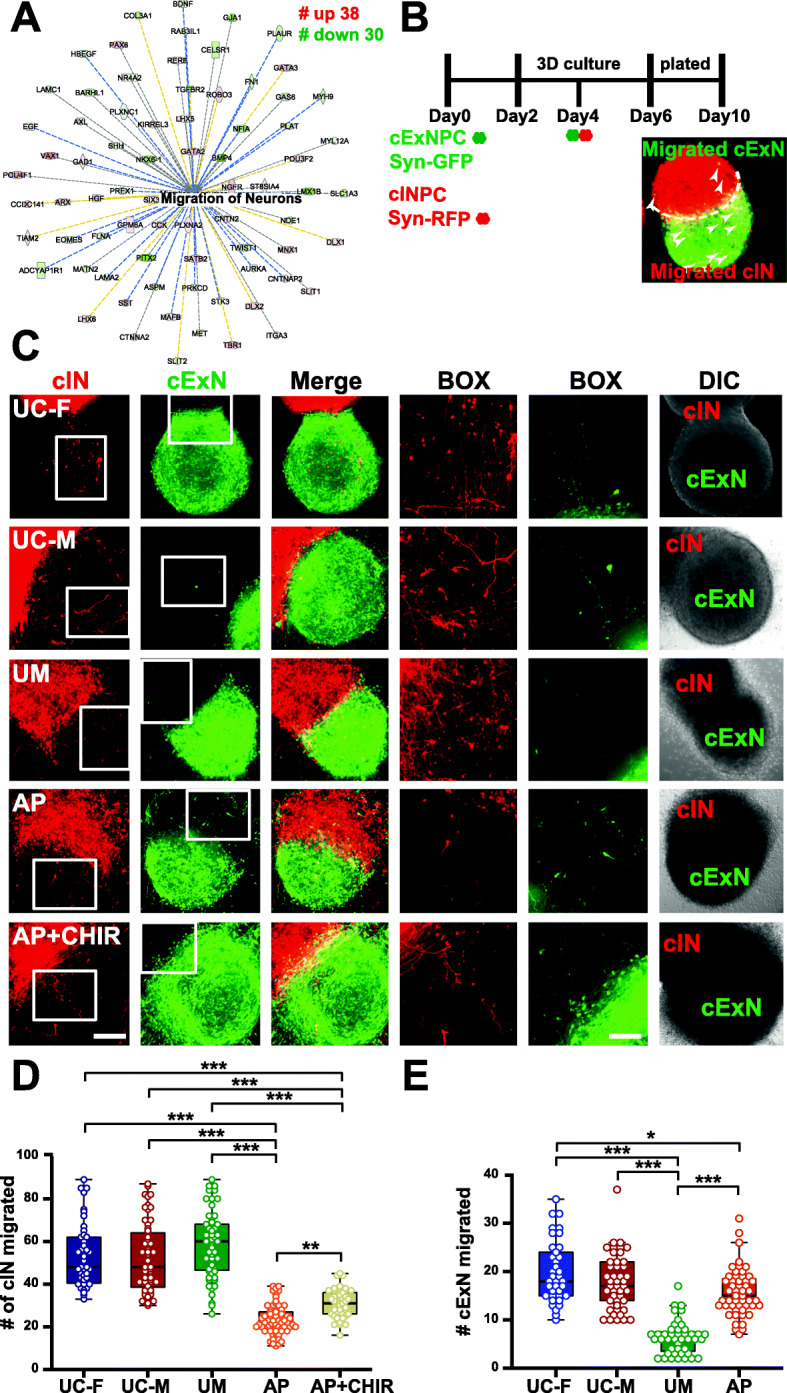
Neuronal migration is compromised in AP-derived cINs and this phenotype is partially reversed by CHIR-99021. **a** IPA analysis of AP-enriched DEGs (versus the UM sample, Fig. 4 above) identified a cluster of genes which regulate neuronal migration. **b** Schematic depicting the migration assay, which involves generating neuroids containing Synapsin promoter (Syn)-GFP-expressing cExNPCs (green) and Syn-RFP-expressing (red) cINPCs, apposition of these neuroids, and differentiation and migration of neurons in these co-cultures, with analysis at day 10. Neurons that migrated into the opposite neuroid are indicated by white arrowheads. **c** Migration of red cINs into the green cExN neuroid, and vice versa, is shown in representative confocal images from assays performed with neuroid co-cultures from all four models. **d** The number of cINs (red) that migrated into the cExN neuroid were quantified from six independent biological replicate experiments (*n* = 6), which used two clonal lines for the UM and AP models and one clonal line for the UC-M and UC-F models. Reduced cIN migration in the AP model was partially reversed by addition of CHIR-99021 (CHIR). **e** Numbers of cExNs (green) that migrated into the cIN neuroid were quantified, using data from six independent biological replicate experiments (*n* = 6) that used two clonal lines for the UM and AP models and one clonal line for the UC-F/UC-M models. Clones used, replicates, and data values are in Additional file 4. Scale bars=150 μm and higher magnification (BOX)=100 μm. *P* values: *P < 0.05, **P < 0.01, and ***P < 0.001 were calculated by using Kruskal-Wallis non-parametric tests as described in the “Methods” section. Plot shows the median value, calculated as described in the “Methods” section

AP-derived neurons (versus the UM) exhibited diminished expression of genes that regulate neuronal migration (Fig. 5a), suggesting that they could have impaired migration. In vivo, cortical interneurons undergo tangential migration from the ventral telencephalon to the cortex. Therefore, we adapted an organoid-based neuronal migration assay (see the “Methods” section): a synapsin-GFP expressing cExN neuroid and synapsin-RFP expressing cIN neuroid were apposed, and neuronal migration from one to the other was evaluated (Fig. 5b). cINs from AP neuroids exhibited reduced migration by comparison with the other models (Fig. 5c-d). By contrast, the migration of AP-derived cExNs was only modestly impaired, while UM-derived cExNs exhibited diminished migration versus the control models, suggesting some impairment of migration in both *CHRNA7* duplication carriers (Fig. 5c, e). As Wnt signaling has been linked to neuronal migration [45], we hypothesized that diminished expression of Wnt signaling-related genes (Fig. 4e) could contribute to the AP model’s reduced cIN migration. Assessment of the AP vs. UM DEGs with the Percayai CompBio tool likewise revealed a network of differentially expressed genes linked to both Wnt signaling and neuronal migration (Additional file 6C-D), some of which (e.g., *GJA1/TGFBR2*) were also identified as Wnt- and neuronal migration-related DEGs in Ingenuity Pathway Analysis (Figs. 4e, 5a). These data suggested that deficits in Wnt signaling could contribute to reduced migration of the AP-derived neurons. Indeed, treatment of AP neuroids with the Wnt agonist CHIR-99021 partially reversed their impaired cIN migration (Fig. 5c-d). However, as a number of other pathways implicated in neuronal migration were also dysregulated in the AP, including axonal guidance, actin cytoskeleton, integrin, and netrin signaling (e.g., Additional file 6), this dysregulation could also have contributed to the deficits in neuronal migration observed in the AP model.

### Increased ER stress selective to the AP-derived NPCs is rescued by the ryanodine receptor antagonist JTV-519

**Fig. 6:**
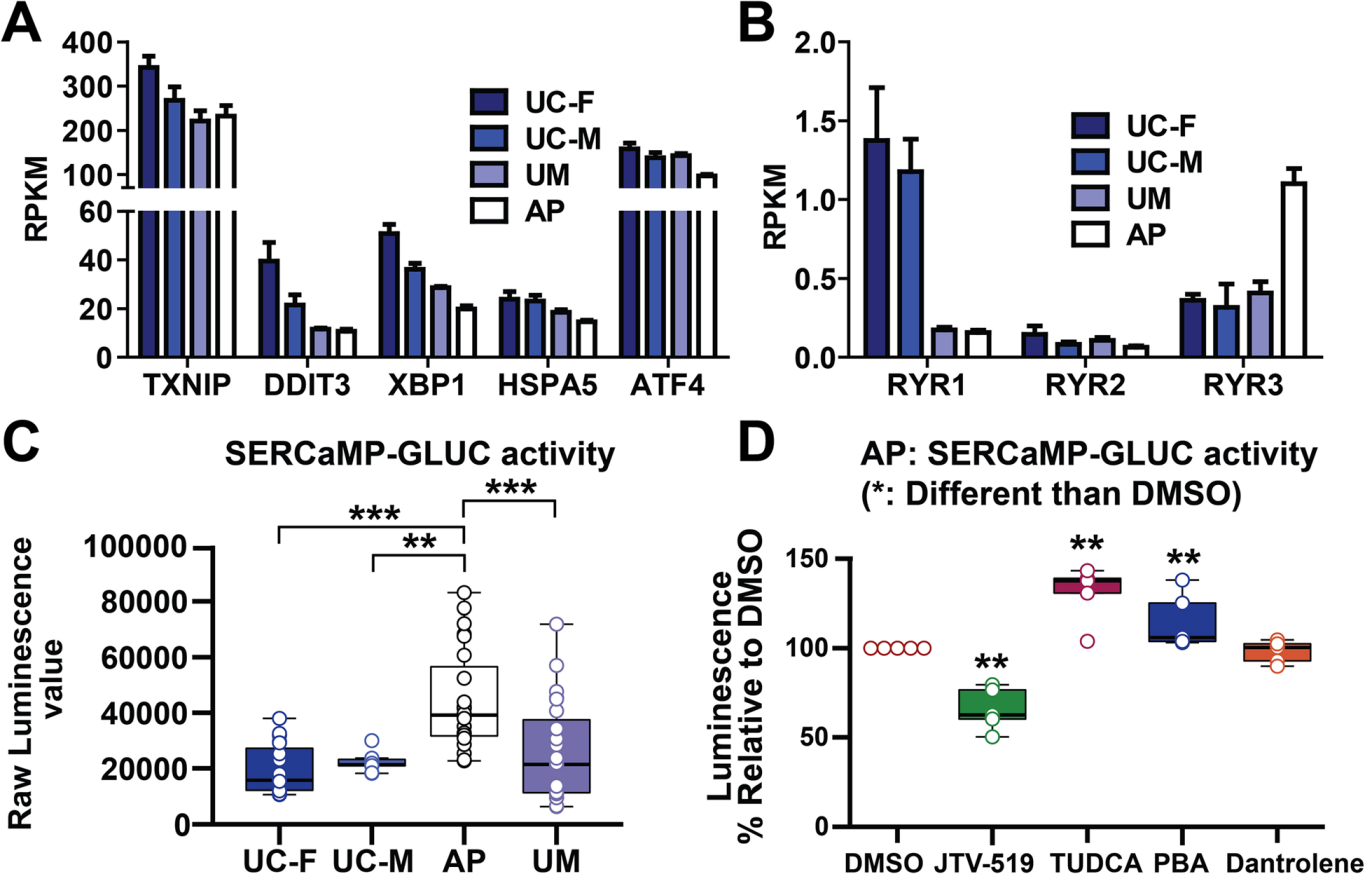
AP-derived cINPCs exhibit elevated ER stress, which is rescued by the ryanodine receptor antagonist JTV-519. **a**, **b** Analysis of differential gene expression between the models did not identify a significant difference in expression of ER chaperones or ER stress genes in **a**, while **b** AP-derived cortical neuroids exhibited increased expression of the neural-enriched ryanodine receptor RYR3. RNA-seq data is derived from four biological replicate experiments (*n* = 4), which used one clonal line per model as described in the “Methods” section. Clones, replicates, and data values are in Additional file 5. **c** Expression of a Secreted ER Calcium Monitoring Protein Gaussia Luciferase stress sensor (SERCaMP-GLUC) in cINPCs demonstrated elevated ER stress in AP-derived cINPCs. **d** The use of the SERCaMP-GLUC reporter assay in the AP model demonstrated that this elevated ER stress phenotype could be suppressed by treatment with the ryanodine receptor antagonist JTV-519, but not with the chemical chaperones PBA and TUDCA or with another ryanodine receptor antagonist, dantrolene. Four independent biological replicate experiments (*n* = 4) were performed using two clonal lines for the AP and UM models and one clonal line for the UC-F and UC-M models. Clones, replicates, and data values are in Additional file 4. Plot shows the median value, calculated by using a Kruskal-Wallis non-parametric test as described in the Methods. *P* values: *P < 0.05, **P < 0.01, and ***P < 0.001

In prior work, microduplication at chromosome 15q13.3 increased CHRNA7 expression and caused moderately increased expression of several endoplasmic reticulum (ER) chaperone and unfolded protein response (UPR)-related ER stress markers [31]. We assessed whether our models likewise exhibited altered expression of ER chaperones in neuroids, but did not observe increased expression of these or other ER chaperone or UPR-related genes in AP-derived samples versus the UM and UC-M models (Fig. 6a; not obtained as DEGs in Additional file 5). However, the ryanodine receptor RYR3, which is most highly expressed in the brain, exhibited increased expression in the AP versus the other samples (Fig. 6b). As ryanodine receptors modulate calcium homeostasis following increased ER stress, we hypothesized that the AP model’s elevated ER stress could involve altered calcium homeostasis, rather than elevated ER chaperone or UPR pathway activities. To assess ER stress in these models, we introduced an expression construct encoding a Secreted ER Calcium Monitoring Protein (SERCaMP)-luciferase stress sensor [46], which monitors stress-related calcium release from the ER, into cINPCs (see the “Methods” section). Interestingly, the AP’s cINPCs selectively exhibited increased ER stress, while the UM did not differ from the UC models (Fig. 6c). As ER stress both activates the UPR and triggers calcium release through ryanodine receptors, we tested whether chemical antagonists of either process could rescue the AP model’s elevated ER stress. While neither UPR antagonist (PBA/TUDCA) exhibited rescue activity, one of two ryanodine receptor antagonists tested (JTV-519, but not Dantrolene) suppressed the AP’s elevated ER stress (Fig. 6d).

### Electrophysiological characterization of cortical neurons derived from the AP, UM, and UC-M models

**Fig. 7:**
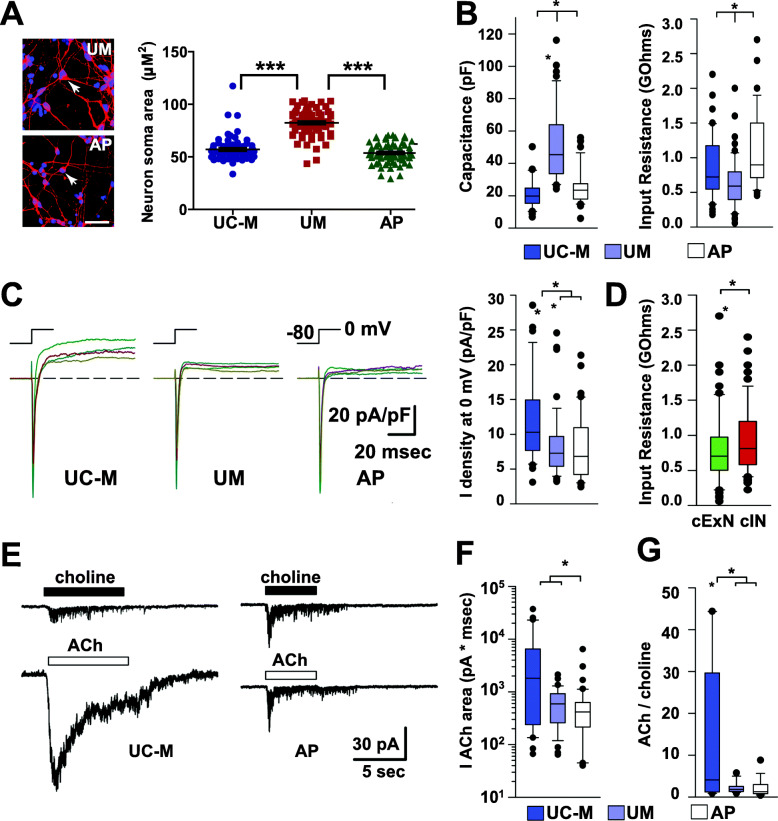
Functional differences in neurons derived from these models were assessed by voltage clamp analysis. **a** Quantitation of neuron soma area revealed a larger soma size in UM-derived neurons. Left: representative confocal images comparing the AP and UM models, with arrows highlighting a cell soma; Right: quantitation of neuron soma area for the three models. Data is derived from three biological replicate experiments (*n* = 3) using two clonal lines for the AP and UM models, and one clonal line for the UC-M model. Data values are in Additional file 4. Plot shows the median value, calculated by using a Kruskal-Wallis non-parametric test as described in the “Methods” section. **b** UM-derived neurons exhibit significantly higher cell capacitance and lower input resistance than UC-M- or AP-derived neurons. **c** Steady-state outward current density at 0mV was significantly greater for UC-M- than for UM- or AP-derived neurons. Whole-cell inward and outward current density for currents were evoked by a voltage step from −80 to 0 mV. **d** Collectively, iPSC-derived cINs from all three subject-derived models exhibited higher input resistance than cExNs. **e** Whole-cell currents were evoked by 500 μM Choline or ACh in neurons, with representative data shown for the UC-M (left) versus AP (right) models. **f** The integrated ACh-evoked current density was significantly smaller for AP-derived neurons and **g** the ACh/choline ratio for integrated current was smaller for both AP- and UM-derived neurons, by comparison with UM-C. Data were derived from three biological replicate experiments (*n* = 3), which used two clonal lines for the AP and UM models, and one clonal line for the UC-M model. Box plots show combined data from cExNs and cINs except in panel (**d**). P <0.05, was determined by 2-way ANOVA on ranks with post hoc Student-Newman-Keuls test. A complete summary of the physiological recordings performed is provided in Additional file 13

**Fig. 8:**
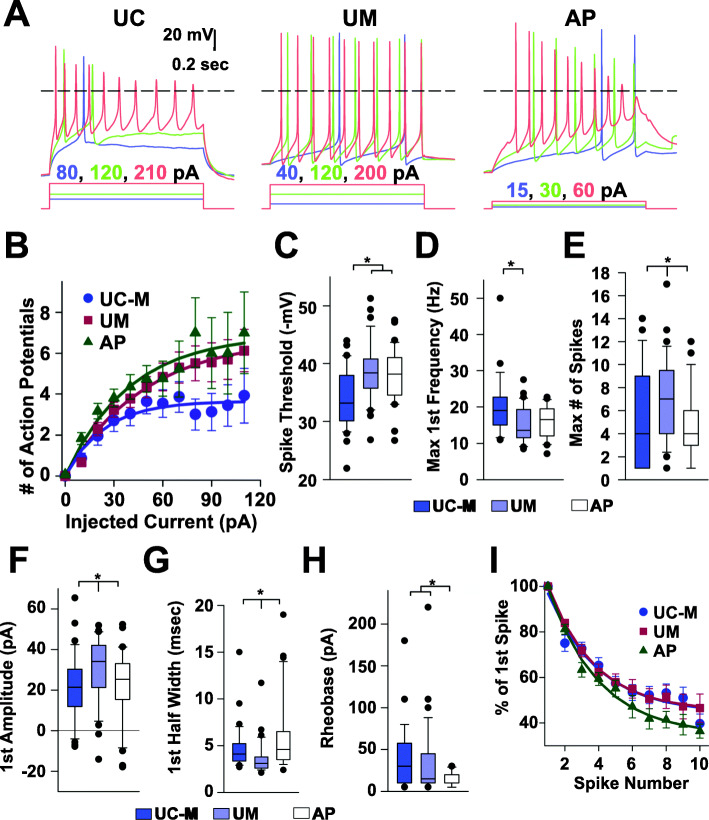
Functional differences in neurons derived from these models were assessed by current clamp analysis. **a**–**c** Action potentials elicited by three different 800 msec depolarizing current injections in iPSC-derived cExNs from the UC-M, UM, and AP models. **b** Exponential fits (smooth curves) indicate significantly fewer action potentials elicited on average by current injections up to 120 pA in neurons derived from the UC-M model, by comparison with the UM and AP models (*p* < 0.05, F-statistic). Recordings under current clamp also revealed **c** a more depolarized threshold for action potential initiation in UC-M-derived neurons compared with both UM- and AP-derived models and **d** a higher initial spike frequency in UC-M compared with UM-derived neurons. **e**–**g** UM-derived neurons exhibited a significantly higher **e** maximal number of spikes and **f** first spike amplitude, and a **G** briefer first spike half-width than UC-M or AP neurons. **h**-**i** Compared to UC-M and UM neurons, AP-derived neurons exhibited **h** a substantially lower rheobase and **I** a more significant decline in average action potential peak amplitude with each succeeding spike (**a** and **i**). Plots show combined data from cExNs and cINs (*n* = 3). P < 0.05 was defined by 2-way ANOVA on ranks with post hoc Student-Newman-Keuls test. A complete summary of the physiological recordings performed is also provided in Additional file 13

To assess whether these models had altered neuronal function, synapsin-GFP-labeled cExNPCs and synapsin–RFP-labeled cINPCs were differentiated as neuroid co-cultures, then were dissociated and grown for 20 days on rat cortical astrocyte feeders to promote neuronal maturation (see Methods; Additional file 12A). Whole-cell voltage and current-clamp recordings were obtained separately from cExNs and cINs from the AP, UM, and UC-M models (18–27 cells assessed/group). For a complete data summary, see Additional file 13. Visual inspection suggested that UM-derived neurons were consistently larger than AP- or UC-M-derived neurons; this was confirmed by cell soma diameter measurements (Fig. 7a; Additional file 12B). This was associated with higher UM cell capacitance (proportional to surface area) and lower input resistance (Fig. 7b). Voltage steps from −80 to 0 mV evoked a fast peak of inward current mediated by tetrodotoxin-sensitive sodium channels, followed by steady-state outward current mediated by voltage-gated potassium channels. Comparisons between models revealed that both the UM and AP had lower outward current density than the UC-M (Fig. 7c). A 2-way ANOVA analysis among all groups also indicated that cINs had a slightly higher input resistance than cExNs (Fig. 7d). Interestingly, although separate recordings were generated and analyzed for cExNs and cINs across the three models, most significant differences were commonly observed in both cExNs and cINs. Therefore, we combined cIN and cExN recording data points for results in Figs. 7 and 8.

Because *CHRNA7* encodes the α-7 nicotinic acetylcholine (ACh) receptor subunit [16], we measured integrated current elicited by brief exposure to 500μM ACh in a subset of cells that were characterized by whole-cell current and voltage clamp. Most of these cells were also tested with 500μM choline, a relatively selective agonist for homomeric α7 receptors [47] with much weaker activity at heteromeric α7β2 receptors or other neuronally expressed nicotinic receptors [48, 49]. Interestingly, UC-M neurons exhibited a significantly larger response to ACh than AP cells (Fig. 7e-f, note log scale in F) and a larger ACh to choline ratio compared to both UM and AP neurons (Fig. 7g). These data suggest that homomeric α7 receptors may mediate a higher proportion of the total ACh-evoked current in both UM and AP neurons, relative to UC-M neurons.

Current clamp recordings (Fig. 8) revealed additional functional alterations shared by UM- and AP-derived neurons. UM and AP cells fired a larger numbers of action potentials on average than UC-M cells, for current injections up to 120 pA (Fig. 8a, b), and displayed a less depolarized threshold for action potential initiation (Fig. 8c). There was also a trend for lower initial spike frequency that reached significance for the UM model (Fig. 8d). In addition to these shared alterations, UM and AP neurons exhibited several functional differences: UM neurons fired a larger maximal number of spikes during an 800 msec depolarizing pulse than UC-M or AP neurons (Fig. 8e) and also exhibited a larger peak amplitude and shorter half width to the first spike than UC-M or AP neurons (Fig. 8f-g). AP neurons uniquely exhibited a substantially lower rheobase (the minimal depolarizing current required to reach threshold; Fig. 8h) and a more significant decline in average action potential peak amplitude with each succeeding spike (Fig. 8a, i), versus both UM and UC-M models. Therefore, by contrast with AP-specific neurodevelopmental phenotypes, the AP and UM models shared a number of functional anomalies not seen in the UC-M model, while also exhibiting some unique characteristics.

Spontaneous excitatory and inhibitory synaptic currents (Additional file 12C) were detected in recordings from all three models; however, only a subset of recorded cells displayed synaptic events and the frequency was very low in all but a few cells. This result demonstrates that, while both cExNs and cINs of all three genotypes are able to form functional connections, the number of fully functional contacts is likely to be low, even after 20+ days of maturation on astrocyte feeder cells.

## Discussion

### Cellular correlates of differential phenotypic penetrance among carriers of *CHRNA7* duplication

Like many CNVs involving microduplication, duplications at 15q13.3 cause multiple psychiatric phenotypes, with patients exhibiting widely varied clinical phenotypic penetrance. How duplication of *CHRNA7* alone versus other genes in this interval causes these phenotypes, and the basis of their variable penetrance, were largely uncharacterized. Therefore, here we derived iPSC models from a pedigree where multiple individuals carried the same CNV, involving only *CHRNA7* duplication, but had differential diagnoses. We compared clonal iPSC models from the UM, who has no clinical phenotypes, her son (AP), who has multiple clinical neurodevelopmental phenotypes, and controls lacking this duplication, using cExN and cIN derivation to examine neurodevelopment and function. This study design enabled the identification of potential contributors to differential phenotypic penetrance between models with the same *CHRNA7* duplication on a partially shared genetic background. While both the AP and UM had elevated CHRNA7 expression, the AP model exhibited many neurodevelopmental phenotypes not seen in the UM, UC-M, or UC-F models. These included reduced neurite extension and length, diminished cIN neuronal migration and formation of VGAT-expressing synaptic punctae, elevated ER stress, and correspondingly reduced gene expression in related molecular pathways and processes. Intriguingly, many pathways with reduced expression in the AP instead exhibited increased expression in the UM model, versus both the AP and controls. This elevated molecular signaling could have compensated for some consequences of the genetic liability of *CHRNA7* duplication, enabling the UM to overcome neurodevelopmental impairment that likely contributed to the AP’s clinical phenotypic penetrance. However, electrophysiological analyses of cExNs and cINs matured on astrocyte feeder layers revealed functional anomalies shared by AP- and UM-derived neurons versus controls, including increased action potential firing and enhanced choline responsiveness. These functional changes are consistent with elevated CHRNA7 channel activity and altered ligand sensitivity, and indicate common functional anomalies likely conferred by *CHRNA7* duplication in both AP- and UM-derived neurons; these, if also manifested in vivo, are nonetheless insufficient to trigger clinical phenotypes in the UM.

This study generally illustrates that patient-derived neuronal models can define cellular and molecular anomalies associated with, and potentially contributing to, differential clinical phenotypic penetrance. Although many CNVs involving microduplication likewise exhibit highly variable phenotypic penetrance, the basis of this phenomenon is not well understood. Here, we distinguished both contributors to differential phenotypic penetrance and those common to multiple carriers of the same genetic liability, regardless of the clinical phenotype. We could also identify changes in molecular signaling that may have compensated for this genetic liability, contributing to a lack of clinical phenotype in the UM. This work indicates that patient-derived iPSC models may have general utility for linking both genetic liabilities and differing phenotypic penetrance stemming from those liabilities to specific cellular, molecular, or functional alterations that may be prior or ongoing contributors to clinical phenotype. These phenotypes then provide a basis and experimental platform for identifying molecular and pharmacological agents that can rescue diagnosis-associated phenotypes, as we did in identifying distinct pharmacological agents that could rescue both the AP model’s ER stress and impaired cIN migration.

### Shared functional alterations among models with *CHRNA7* duplication

Functional assessment of neurons differentiated from these models revealed a number of electrophysiological abnormalities common to both individuals with *CHRNA7* duplication (AP and UM), regardless of clinical diagnosis. The CHRNA7 receptor forms either homomeric or heteromeric pentamers; these exhibit differential responsiveness, with homomeric α7 receptors having selective choline sensitivity, while heteromeric receptors are more ACh responsive [47]. Here, both AP- and UM-derived neurons exhibited elevated choline responsiveness and diminished ACh responsiveness, versus controls. This is congruent with the possibility that *CHRNA7* duplication, which increased CHRNA7 expression in the AP and UM, may increase the production of homomeric α7 channels, altering electrophysiological function and ligand sensitivity. Other functional abnormalities were also shared by UM- and AP-derived neurons, versus controls, including a reduced outward potassium (K) current and increased number of action potentials. Together, these results suggest that increased channel activity may enhance entry of calcium, sodium, and potassium ions into neurons, increasing action potential firing in both models with 15q13.3 duplication.

### Altered developmental mechanisms that track with the AP model’s severe phenotype

Congruent with a prior study [31], we observed elevated ER stress in iPSC-derived NPCs carrying *CHRNA7* duplication in the AP but not the UM model. However, the ER stress phenotypes observed in these two studies differ substantially. Nicotinic acetylcholine receptor trafficking in cells involves assembling five subunits into a pentameric receptor in the endoplasmic reticulum (ER), followed by trafficking to the plasma membrane [50, 51]. In Gillentine et al., elevated ER stress was attributed to the unfolded protein response (UPR), based upon increased expression of several ER chaperone- and UPR-related marker genes [31]. By contrast, our mRNA expression analysis did not reveal altered expression of these or other UPR-related markers in the AP. However, altered calcium homeostasis is another aspect of ER stress [52]: to maintain homeostasis, the ER releases calcium via several mechanisms, one of which involves ryanodine receptor signaling activation in response to altered cytoplasmic calcium, through calcium-induced calcium release (CICR) [53]. Our analysis revealed elevated levels of both calcium receptors CACNA1A, CACNA1B, and CACNA2D2, and RYR3, a ryanodine receptor with brain-enriched expression [53], in the AP, suggesting potentially altered calcium homeostasis. Accordingly, a reporter assay detected elevated ER stress-linked CICR in the AP, versus the other models, and the ryanodine receptor antagonist JTV-519 selectively reduced this response to baseline conditions, while UPR antagonists did not. These results further implicate altered calcium homeostasis rather than UPR pathway activation as the major contributor to the AP model’s elevated ER stress here. Another major difference between these findings is that the NPCs in Gillentine et al. exhibited characteristics consistent with reduced CHRNA7 protein trafficking to the cell membrane and reduced CHRNA7 channel activity. By contrast, when we conducted electrophysiology on neurons from our models after maturation on astrocyte feeder layers, both *CHRNA7* duplication carriers (AP and UM) displayed hallmarks of increased CHRNA7 channel activity, including increased action potential firing, and altered ligand sensitivity. Therefore, although both studies observed elevated ER stress in models with *CHRNA7* duplication, here reporter assays and pharmacological rescue demonstrated that, for AP-derived models, this involved altered calcium homeostasis rather than the UPR, and that *CHRNA7* duplication in the AP and UM increased, rather than diminished, channel activity. It will be interesting to assess these phenotypes in additional models with *CHRNA7* duplication in future work, to determine whether effects on ER stress and channel function indeed vary between models derived from different subjects; these could relate to other aspects of particular models, such as interactions with the patient’s genetic background, specific clinical phenotypes, or degree of phenotypic penetrance.

In this study, the AP model exhibited many neurodevelopmental anomalies, including increased NPC proliferation but reduced neurite outgrowth and length, and concordant dysregulation of genes mediating axonal guidance, gap junctional connectivity, and neuritogenesis, with reduced expression of most genes in these pathways in the AP versus the UM and controls (Additional file 14 [54–65];). Altered NPC proliferation, neuronal differentiation, neurite extension, neuronal maturation, and synapse formation have also been observed in other iPSC models derived from ASD patients [18–20, 23, 27, 54, 66–68] and in clinical studies of ASD and other neuropsychiatric disorders [54–58]. These findings are intriguing since the AP and UM share the same *CHRNA7* duplication, but only the AP exhibits these neurodevelopmental alterations, which may contribute to his pathogenesis. UM-dysregulated genes were linked to many similar GO terms, including axonal guidance, integrin and gap junctions, and behavior- and nervous system development; however, these were often upregulated in the UM versus both AP and UC models, suggesting compensatory molecular changes that may have normalized some corresponding neurodevelopmental processes in the UM, potentially contributing to an absence of clinical phenotypes. In contrast to these AP-specific neurodevelopmental deficits, electrophysiological analysis of more mature cells revealed functional anomalies that were common to both AP and UM cells, raising the possibility that maturation or the influence of astrocyte feeder cells may modify the phenotypic relationships among the UM-C, UM, and AP models.

Altered GABAergic neurotransmission is implicated in ASD affectation and may result from disrupted synaptic development, which can imbalance excitatory and inhibitory neuronal activity in the cortex [33, 69]. Most in vitro modeling studies characterize neurodevelopmental phenotypes only in excitatory, glutamatergic neurons [18, 27, 70, 71]; therefore, the extent to which GABAergic neurodevelopment is altered in ASD iPSC models remains largely unexplored. Here, we observed multiple phenotypes only in AP-derived cINs but not cExNs, suggesting that assessing both neuronal types may enable the identification of additional cell-type-specific potential contributors to disease. For example, we observed the reduced formation of VGAT-expressing punctae in AP-derived cINs, which could alter cIN function, potentially impairing synaptic activity to disrupt excitatory-inhibitory balance in the AP. We did not attempt to quantify a difference in functional connectivity owing to the low overall prevalence of spontaneous synaptic activity in all three of the models at the time point evaluated; however, this could be a promising topic for future study. In the cortex, inhibitory neurons constitute a minority population (~20%) [72, 73], are specified in sub-cortical progenitor territories, and undergo tangential migration to their cortical targets. Migration of these and other neurons is critical for normal neurodevelopment [74, 75]. Accordingly, here, the AP exhibited decreased cIN migration and diminished expression of corresponding suites of genes that regulate migration (Fig. 5a; Additional file 14). Other studies of ASD and other neuropsychiatric disorders have likewise revealed evidence of abnormalities in neurogenesis, neuronal migration, and neuronal maturation [2, 61, 76, 77]. Furthermore, Wnt signaling contributes to neuronal migration, and here many Wnt signaling genes had reduced expression in the AP model, while the Wnt agonist CHIR-99021 partially rescued this model’s migration deficit (Figs. 4e, 5; Additional file 14). Modulating Wnt signaling has been explored for ASD treatment; however, as both reduced and elevated Wnt activities are implicated in ASD-related behavioral and cognitive alterations, further studies are required to stratify how Wnt pathway biomarkers are altered in neuropsychiatric disorders including ASD [63–65].

### Limitations

This work assessed how *CHRNA7* duplication affects neurodevelopment and function and identified potential contributors to variable phenotypic penetrance among 15q13.3 duplication carriers. As the study design involved comparing first-degree relatives with the same duplication but differential diagnoses, age, and sex represent potential confounds for data interpretation. ASD is more prevalent in males than females in the population, with evidence suggesting that females may require a higher load of genetic liability to exhibit clinical phenotypes [78]. However, here the relationship of differential penetrance to sex differences is unclear, as 15q13.3 is autosomal rather than sex chromosome-linked, and current iPSC modeling approaches cannot recapitulate random X-chromosome inactivation that female somatic tissues, including the brain, undergo [79, 80]. However, in our RNA-seq analysis, very few (<3%) DEGs were either X-chromosome-linked or exhibit sex-biased expression in the human brain [81, 82]. Age is another unavoidable confound in studies involving modeling multiple subjects in the same pedigree. It is currently unclear whether iPSC lines derived from old versus young donors exhibit differences in their potential for differentiation or senescence, and this issue is controversial [83, 84]. To address these confounds, we performed variancePartition analysis, which indicated that differences between samples were predominantly driven by subject identity and genetic background, while age and sex were minimal contributors to DEG identification. Another confounding variable in many iPSC modeling studies is differences in genetic background. Here, the AP and UM have a partially shared genetic background, including the same 424kb duplication at 15q13.3. The large size of this duplication precludes CRISPR-mediated correction, which could enable the identification of duplication-linked phenotypes on an isogenic background. Finally, we could not access biological material from the father, who represents an unaffected male first-degree relative control. As 50% of the AP’s genome derives from the father, it would have been interesting to understand whether additional paternally inherited genetic liabilities could have contributed to the AP's phenotypic penetrance. However, biomaterials were not available for study from this subject. Despite these limitations, this work still identified cellular, molecular, and functional signatures that differed in clinically affected versus unaffected individuals carrying the same contributory genetic duplication on a partially shared genetic background, as well as defining functional alterations shared by both *CHRNA7* duplication carriers, that distinguished them from unrelated controls lacking *CHRNA7* duplication.

## Conclusions

It is challenging to identify variables that contribute to the wide variability of clinical phenotypic penetrance among individuals carrying microduplications in the same genomic region. These anomalies usually cannot be recapitulated in animal models, confounding experimental study, while both the genomic interval, which genes are involved, and heterogeneous genetic background contributors in unrelated individuals can confound iPSC model cross-comparison. Here, using related individuals with the same duplication but variable phenotypes was informative in identifying neurodevelopmental deficits and elevated ER stress specific to AP-derived models and potentially contributing to his clinical diagnoses. Conversely, upregulation of overlapping developmental signaling pathways in the UM may have contributed to a lack of both neurodevelopmental phenotypes in this model and clinical diagnoses in this subject. Finally, despite their variable diagnoses, both models carrying *CHRNA7* duplication exhibited shared functional abnormalities. Therefore, these findings highlight the potential for iPSC models to identify cellular and molecular anomalies linked to either the presence versus absence of a psychiatric disorder-linked CNV, such as 15q13.3 duplication, and to differences in phenotypic penetrance among multiple individuals carrying the same CNV.

It would be interesting to examine whether neurodevelopmental deficits, particularly in cINs, are associated with patient phenotype in models from other *CHRNA7* duplication carriers, to determine whether the phenotypes identified here frequently predict clinical diagnoses in this patient population. It would also be intriguing to know whether these phenotypes are more broadly generalizable indicators of phenotypic penetrance in other CNVs involving genome microduplication, many of which exhibit similarly variable clinical phenotypic penetrance. Defining general hallmarks of and contributors to phenotypic penetrance in CNVs involving microduplication, at either 15q13.3 or other susceptible genomic intervals, would be informative in defining anomalies that contribute to these disorders, while these iPSC models provide a platform for molecular and pharmacological screening for phenotypic rescue, to develop interventions for treating affected individuals.

## Methods

### Clinical phenotypes

Clinical phenotypes of these three subjects with 15q13.3 duplication are summarized in Table 1. The AP’s pregnancy was planned and there was no history of in utero exposure to drugs, alcohol, or tobacco. There were no complications during the pregnancy, and the AP was delivered normally at full term, with a birth weight of seven pounds, six ounces. As an infant, the AP was delayed in sleeping through the night, which did not begin to occur until after 1 year of age. He has no history of delay in motor development but has a history of significant delay in language development, producing his first word after the age of 2 years. He started speech therapy at 2 years of age, resulting in rapid improvement in his language development. The AP was 12 years old when this study was initiated, with a history of ADHD, depression, and ASD. Prior to age five, he reportedly did not make eye contact and did not exhibit age-appropriate social reciprocity. While in the third grade, the AP had frequent crying episodes and was overwhelmed by homework, which brought him to psychological evaluation. At that time, he manifested both autistic features and pronounced mood lability on exam, which was manifested on several occasions by the child becoming morose and tearful when told even slightly sad stories that would not have elicited such a reaction in a typical child his age. During periods of depressive symptomatology, his mother reported that he had difficulty falling asleep, low appetite, and decreased interest in his favorite activities, such as sports. In middle school, he manifested poor concentration and difficulty with time management, standardized test-taking, and organizing tasks and activities. These issues were treated by using cognitive behavioral therapy and play therapy. He was subsequently treated with sertraline, followed by escitalopram. On these selective serotonin reuptake inhibitors, the AP’s anxiety was significantly lessened, but he then experienced a residual lack of motivation and his perseverative traits were not improved by treatment. Based on his developmental history, including language delay, impairment in social reciprocity and non-verbal communication, and repetitive thinking, it was clinically determined that a significant contribution to his overall impairment was autistic perseveration and rigidity, for which a trial of risperidone was initiated and resulted in significant clinical improvement over the ensuing years. Ultimately, he was successfully weaned from risperidone and was reasonably well adapted in high school. The AP’s mother reported a history of mild depression, anxiety, and obsessive-compulsive traits, while the AP’s 8-year-old brother had subtle autistic traits that were less pronounced than those of the AP and behavioral features of emotional dysregulation that were more pronounced than those of the AP; he met criteria for disruptive mood dysregulation disorder, ADHD, and mood disorder.

### Genotyping

Cytogenetics Microarray (CMA) analysis was performed for research testing by the Washington University Cytogenetics and Molecular (CMP) Pathology Laboratory, using the Affymetrix CytoScan HD array. This array includes 2.6 million copy number markers, 1.9 million non-polymorphic probes, and nearly 750,000 single-nucleotide polymorphism (SNP) probes. Average intragenic marker spacing is equivalent to 1 probe per 880 basepairs. Analysis of these data by the CMP Laboratory, after alignment to hg19, defined a 424 kb gain at 15q13.3 in samples from the affected proband (AP) and unaffected mother (UM) and a 444 kb gain in the same location in the affected brother. This copy number variant was not present in the father.

### iPSC generation

The Washington University Genome Engineering and Induced Pluripotency Center (GEiC) derived multiple clonal iPSC lines from individuals in this family. Briefly, fresh urine samples were procured from the AP and UM and were used to obtain renal epithelial cells, which were reprogrammed using the CytoTune-iPSC 2.0 Sendai virus-based reprogramming kit (Thermo Fischer Scientific), as per the manufacturer’s instructions. iPSC clones were picked and three clonal cell lines were derived from each study subject. Clones number 1 and 3 from each subject were used for experimentation. These newly derived lines were compared with established male and female iPSC control lines from unrelated individuals (BJFF6 and AN1.1, denoted UC-M and UC-F in this study) provided by the Washington University Genome Engineering and Induced Pluripotency Center.

### iPSC cultures and differentiation

iPSC line derivation and directed differentiation were performed by modification of our previously described methods [22, 85]. iPSC lines were grown on Matrigel (Corning) under feeder-free conditions using mTeSR1 (STEMCELL Technologies), authenticated by STR profiling, and tested for mycoplasma contamination regularly during culture. For directed differentiation to generate cortical excitatory neural progenitor cells (cExNPCs), iPSCs were dissociated into single cells with Accutase (Life Technologies) and 40,000 cells were seeded in V-bottom 96 well non-adherent plates (Corning). Embryoid bodies (EBs) were generated by centrifugation of the plate at 200xg for 5 min and were then incubated in 5% CO_2_ at 37°C, in cExNPC differentiation medium with 10μM Y-27632 (Tocris Biosciences). cExNPC differentiation medium includes Neurobasal-A (Life Technologies), 1X B-27 supplement without Vitamin A (Life Technologies), 10μM SB-431542 (Tocris Biosciences), and 100nM LDN-193189 (Tocris Biosciences). On day 4, EBs were picked with wide bore P1000 tips and were transferred to Poly-l-Ornithine- (20μg/ml) and laminin- (10μg/ml) coated plates. Every other day media without Y-27632 was replenished, and on day 15 Neural Rosette Selection Reagent (STEMCELL Technologies) was used to isolate cExNPCs from rosettes, as per the manufacturer’s instructions. cExNPCs were grown as a monolayer using cExNPC differentiation media up to 15 passages.

Cortical inhibitory neural progenitor cells (cINPCs) were generated by directed differentiation in media which included Neurobasal-A (Life Technologies), 1X B-27 supplement without Vitamin A (Life Technologies), 10μM SB-431542 (Tocris Biosciences), 100nM LDN-193189 (Tocris Biosciences), 1μM Purmorphamine (Calbiochem), and 2μM XAV-939 (Tocris Biosciences). Y-27632 was also included in this media until day eight. For cINPC differentiation, EBs were generated as described above for cExNPC differentiation. On day 4, EBs were transferred to non-adherent plates and were placed on an orbital shaker at 80rpm in an incubator with 5% CO_2_ and 37°C. cINPC media were replenished every other day, and on day 10, EBs were transferred to Matrigel- and laminin- (5μg/ml) coated plates. On day 15, cINPCs were dissociated with Accutase and were either cryopreserved and/or grown in monolayer culture on Matrigel- and laminin-coated for up to 15 passages. For analysis of both cExNPC and cINPC growth properties, equal numbers of cells for each line were seeded on Matrigel- and laminin- (5μg/ml) coated plates and the total number of cells was counted after 4 days.

For differentiation and maturation of neurons, cortical neuroids were generated by seeding both 2X10^4^ cExNPCs and 2X10^4^ cINPCs into each well of a V-bottom 96 well non-adherent plate in maturation media. Plates were spun at 200xg for 5 min and were incubated in 5% CO_2_ at 37°C in maturation media, with addition of Y-27632 for the first 4 days of culture. The composition of maturation media includes Neurobasal-A and 1X B-27 supplement without vitamin A, while DAPT (10μM; Tocris) was included in the media from day 7 to day 11, and 200μM ascorbic acid (Sigma Aldrich), 20ng/ml BDNF (Peprotech), and 200μM cAMP were included in the media from day 11 to day 15. On day 4, neuroids were transferred to non-adherent plates and were placed on an orbital shaker at 80rpm in an incubator with 5% CO_2_ and 37°C. On day 5, neuroids were moved to Matrigel- and laminin- (5μg/ml) coated plates and further incubated in 5% CO_2_ at 37°C. Media were replenished every other day until day 15.

### Immunocytochemistry (ICC) and immunoblotting

ICC and immunoblotting experiments were performed as previously described [22]. In brief, for ICC, cortical neuroids were dissociated after 15 days of maturation and cells were plated in eight-well chamber slides coated with Matrigel and laminin (5μg/ml). After 24 h, cells were washed with PBS without calcium and magnesium and were fixed in 4% paraformaldehyde for 15–20 min. See [22] for detailed protocol. Primary and secondary antibodies used for these experiments and for immunoblotting are provided in Additional file 4. Images were taken using a spinning-disk confocal microscope (Quorum) and an Olympus inverted microscope using MetaMorph software. ImageJ was used to process images and for quantification: 15–20 random fields were imaged from three to five biological replicate experiments, which included work with two different clones per sample type for the AP and UM models and one clone for the UC-M/F models; total numbers of both immune-positive and all DAPI stained cell nuclei quantified are shown in Additional file 4. For each experimental finding in this manuscript, the number of biological replicate experiments and the clones used for each replicate of each type of experiment is also summarized in Additional file 4.

### FACS analysis

Approximately one million cExNPCs or cINPCs between passages 4–9 were used for FACS analysis, and experiments were performed as described previously [22]. *The plot shows the median value* gathered from seven biological replicates, using two different clones per sample type for the AP and UM models and one clone for the UC-M/F models, calculated by using a Kruskal-Wallis non-parametric test. *P* values: *P < 0.05, **P < 0.01, and ***P < 0.001.

### RNA-Sequencing and RT-qPCR

After 15 days of cortical neuroid differentiation as described above, total RNA was collected from the AP, UM, UC-M, and UC-F lines, using the NucleoSpin RNA II kit (Takara) per the manufacturer’s instructions. RNA was quantified using a NanoDrop ND-1000 spectrophotometer (Thermo Scientific) and the Agilent Bioanalyzer 2100 was used to assess RNA integrity, with only samples with an RNA Integrity Number of >8 used for sequencing and analysis. RNA-Sequencing (RNA-Seq) library preparation and Illumina Sequencing were performed by the Genome Technology Access Center at Washington University. The Illumina Hi-Seq3000 was used to obtain single-end 50 base pair reads, with approximately 30 million unique reads per sample obtained after alignment. Four independent biological replicates per cell line were analyzed by RNA-Seq. For RT-qPCR, 1μg total RNA was reverse transcribed using iScript Reverse Transcription Supermix (Bio-Rad) and equal quantities of cDNA were used as a template for RT-qPCR using the Applied Biosystems Fast Real-Time quantitative PCR platform. GAPDH or RPL30 were used as endogenous controls for normalization. Four biological replicate experiments using one clonal line for each sample type were used for RNA-seq analysis (*n* = 4), while a second clonal line for the UM and AP models was used to generate RNA for RT-qPCR validation of a subset of the RNA-Seq findings. *P* values for RT-qPCR validation: *P < 0.05, **P < 0.01, and ***P < 0.001 were determined by unpaired t testing.

### Bioinformatics and IPA analysis

RNA-Seq data analysis was performed as described in [22, 85] to obtain differentially regulated genes (DEG). Briefly, STAR version 2.5.4b was used to align the RNA-Seq reads to the human genome assembly hg38 [22]. To derive uniquely aligned unambiguous reads, we used Subread:featureCount, version 1.6.3 with GENCODE gene annotation [22] and gene-level transcripts were imported into the R-bioconductor package [22]. After excluding genes expressed at <1.0 counts per million (CPM), differentially expressed genes (DEG) were curated based upon a Log2 fold change >1 and a Benjamini and Hochberg FDR of <0.05. DEGs were used to perform hierarchical clustering analysis using ClustVis [86] and to perform Ingenuity Pathway Analysis (IPA) (Qiagen), as described previously [22]. To determine the contribution of different covariates to gene expression, we also performed variancePartition analysis, including the individual sample types (UC-M, UC-F, UM, and AP), age (young and old), and sex (male and female) as variables [87].

### Morphometric analysis

To measure neurite extension, cortical neuroids were generated and cultured to promote differentiation and maturation as described above. On day 6, images were acquired using an inverted light microscope, with data collected for three or more independent biological replicate experiments encompassing work with two clonal lines per subject for the AP and UM, and one clonal line for the UC-F and UC-M. Neurite extension length from adherently plated neuroids was measured using ImageJ, as the distance between two circles drawn at the border of the plated neuroid and at the tips of neurites extending from that neuroid. Since neurites extend in all directions from each plated neuroid, each datapoint is a calculation of neurite length from the EB border to the tip of the neurites, calculated on a per-EB basis and normalized by the number of EBs quantified per sample type (e.g., UC-M). The plot shows the median value calculated from 50–80 data points per sample type, with data points gathered from seven biological replicates.

ICC for MAP2 in adherent neuroids was conducted as described above, with image acquisition using a spinning-disk (Quorum) confocal microscope and an Axiovision inverted microscope. Day 15 neuroids were also dissociated and the neurons plated on Matrigel- and laminin-coated plated and stained with MAP2.

To assess neurite length, images were acquired using a spinning-disk (Quorum) confocal microscope and an Axiovision inverted microscope. Images were processed with Imaris software (Bitplane) and neurite length was measured using the filament tracer application and normalized to the number of nuclei stained with DAPI in the neuroid, which was measured with the particle application in Imaris. The neuronal soma area was measured from MAP2 stained images using ImageJ. The data points are normalized on a per neuron basis, with neurons quantified from at least 20 different images (with at least five neurons measured per image) per biological replicate, with a total of three independent biological replicates from two clones analyzed.

To quantify VGAT- and VGLUT-expressing punctae, dissociated and plated neuroids generated as described above were immunostained for the respective antibodies, and images were taken using a spinning-disk (Quorum) confocal microscope and Axiovision inverted microscope. Punctae were measured using the synaptic counter plugin in ImageJ. Each finding was obtained in three or more independent biological replicate experiments encompassing work with two clonal lines per subject for the AP and UM, and one clonal line for the UC-M.

### Migration assay

To study the migration of cExN and cIN neurons, we developed an approach that utilized fused co-culture of two 3D spheres consisting of cExNs and cINs. These 3D spheres were generated by transducing cExNPCs and cINPCs separately with either a lentiviral synapsin-eGFP or a synapsin-RFP expression construct, respectively. 30,000 of these transduced cExNPCs or cINPCs per well were then seeded into separate wells of a V bottom 96 well plate in 100μl of maturation media containing 10μM Y-27632. The V bottom plate was centrifuged at 200xg for 5 min at room temperature and then incubated in 5% CO_2_ at 37°C. On day 2, 50μl of media was replaced with fresh media without disturbing the spheres. On day 4, cExN and cIN EBs were selected with wide bore P1000 tips and moved to a U bottom plate. One cExN and one cIN sphere were placed side by side in each U bottom well. Placement of spheres in close apposition caused them to undergo fusion without further manipulation, enabling assessment of neuronal migration. On day 6, these fused spheres were moved with wide bore P1000 tips to a coverslip placed in a 3-cm plate and coated with matrigel and laminin (5μg/ml). On day 10, images were acquired using a spinning-disk (Quorum) confocal microscope and Axiovision inverted microscope and image processing was performed using ImageJ. Migration was assessed in such fused co-cultures. The ability of Wnt signaling to rescue migration deficits observed in the AP line was tested by the addition of 10μM CHIR-99021 in DMSO, with parallel treatment of control spheres with equal quantities of DMSO. Three or more independent biological replicate experiments were performed in two clonal lines for the AP and UM, and in one clonal line for the UC-F and UC-M. Each data point is the number of neurons that migrated from the cIN into cExN EB or from the cExN into the cIN EB, normalized to each fused EB (e.g., EB pair). Each data point represents the number of migrating cExNs per fused EB or the number of cINs per fused EB, with the median value calculated from ~40–80 fused EBs per sample type. The plot shows the median value calculated from 40 to 80 data points per sample type, with data points gathered from six biological replicates, using two different clones per sample type. *P* values were calculated by using a Kruskal-Wallis non-parametric test. *P < 0.05, **P < 0.01, and ***P < 0.001.

### ER stress luciferase assay

To test the effects of endoplasmic reticulum (ER) stress on cINPCs, we used an expression construct encoding a stress sensor [46]. This construct encodes a Guassia luciferase protein fusion, with replacement of the first 18 amino acids with the signal peptide from the mesencephalic astrocyte-derived neurotrophic factor (MANF) protein and carboxy-terminal fusion to MANF’s final 5 amino acids, which encode a stress sensor. To detect ER stress, 35,000 cINPCs were seeded on Matrigel- and laminin- (5μg/ml) coated 96 well plates in cIN differentiation media containing Y-27632. After 24 h, the cells were transfected with the stress sensor expression construct using FuGENE 6 (Promega) transfection reagent. 48 h after transfection, 50μl of supernatant was removed and assayed for luciferase activity using the BioLux Gaussia Luciferase Assay Detection System (New England Biolabs). For rescue experiments, small molecules were obtained from Sigma Aldrich or Tocris Biosciences, reconstituted in DMSO or PBS-Ca^2+^/Mg^2+^, and added in the medium after 24 h of transfection, at the final concentrations indicated (Tudca-50μM, PBA-500μM, Dantrolene sodium-1μM, and JTV-519-10μM). Luciferase levels were measured after 48 h of small molecule treatment as above. Luciferase data for rescue experiments performed in the presence of small molecules were normalized to the DMSO control, and three or more independent biological replicate experiments were performed, using two clonal lines for the AP and UM and one clonal line for the UC-F and UC-M.

### Electrophysiology

cExNPCs and cINPCs were transduced with Synapsin-GFP and Synapsin-RFP expression constructs, respectively, before performing cortical neuroid maturation. At day 0 of cortical neuroid differentiation, 2X10^4^ cExNPCs and 2X10^4^ cINPCs were mixed in each well of a V-bottom 96-well non-adherent plate in maturation media. Maturation followed the approach described above. At day 15, neuroids were dissociated using Accutase (Life Technologies), were seeded onto a layer of rat cortical astrocytes, prepared as described previously [85], and were grown for another three weeks using Neurobasal-A, 1XB27 with vitamin A (Life Technologies), and supplementation with BDNF (20ng), cAMP (200μM), and ascorbic acid (200μM). iPSC-derived neuron cultures were perfused at 1 ml/min with room temperature (22°C) Tyrode’s solution (in mM): 150 NaCl, 4 KCl, 2 MgCl2, 2 CaCl2, 10 glucose, 10 HEPES, with pH adjusted to pH 7.4 with NaOH. Recording electrodes had an open-tip resistance of 2–6 MOhm when filled with (in mM): 140 K-glucuronate, 10 NaCl, 5 MgCl2, 0.2 EGTA, 5 Na-ATP, 1 Na-GTP, and 10 HEPES, pH adjusted to 7.4 with KOH. Whole-cell currents and membrane potentials were recorded with an Axopatch 200A amplifier (Molecular Devices). Voltage clamp recordings were used to determine cell capacitance and input resistance as well as peak inward sodium current and steady-state outward potassium currents during depolarizing voltage steps from a holding potential of −80 mV [85]. Current clamp recordings and currents evoked by choline and acetylcholine (ACh) were obtained in a modified extracellular solution (in mM): 120 NaCl, 3 KCl, 10 glucose, 1 NaH_2_PO_4_, 4 NaHCO_3_, 5 HEPES, pH adjusted to 7.4 with NaOH, delivered from an 8-barrelled local perfusion pipette positioned very close to the recorded cell in order to minimize desensitization. Although desensitization can be further reduced by recording from outside-out patches that enable faster solution exchange, the present study employed whole-cell recordings in order to allow for current and voltage-clamp analysis of cellular physiology as well as agonist-gated currents all within the same cells. In future experiments, higher speed agonist delivery would allow for a more detailed kinetic comparison among the genotypes.

### Statistical analysis

All statistical analyses were performed using IBM SPSS Statistics (v.27) or Sigma-STAT. Prior to analyses, data was screened for missing values and fit of distributions with assumptions underlying univariate analysis. This included the Shapiro-Wilk test on *z*-score-transformed data and qqplot investigations for normality, Levene’s test for homogeneity of variance, and boxplot and *z*-score (±3.29) investigation for identification of influential outliers. Means and standard errors were computed for each measure. Non-parametric Kruskal-Wallis and Mann-Whitney U tests were used to analyze data. The critical alpha value for all analyses was *P* <0.05, unless otherwise stated. Multiple pairwise comparisons were subjected to Bonferroni correction, where appropriate. The datasets generated and analyzed during the current study are available from the corresponding author upon reasonable request.

## Supplementary Information

**Additional file 1.** Characteristics of subjects in pedigree modeled here. (.jpg). **(A)** The study samples were derived from a pedigree with 15q13.3 duplication, with differential clinical affectation indicated by shading of subjects. The affected proband (AP) is represented in black, his affected brother, shown in gray, exhibits subtle autistic traits and has more volatile emotional dysregulation than the AP, while the unaffected mother (UM) and father are shown in white. Renal epithelial cells from the family members indicated (*) were used to derive iPSC models. **(B)** CNV array data for the AP and UM shows the signal intensity (log2 weighted ratio) and predicted copy number across the duplicated region of 15q13.3. The region lacking signal was not covered by the CNV array used for this analysis.

**Additional file 2.** Characterization of iPSC models. (.jpg). Renal epithelial cell-derived iPSC lines from the UC-M, UM, and AP subjects **(A)** exhibit normal human stem cell colony morphology in bright field images (scale bar = 250 μm), **(B)** express the pluripotency marker OCT4/POU5F1 (scale bar = 150 μm), and **(C)** have a normal karyotype.

**Additional file 3 **Cell cycle analysis of neural progenitor cells (NPCs). (.jpg). **(A)** cExNPCs and **(B)** cINPCs were stained with propidium iodide for DNA content and analyzed by FACS. Percentages of cells in each phase of the cell cycle were quantified for each model. Values shown are from seven independent biological replicate experiments (*n* = 7), using two clonal lines for the UM and AP, and one clonal line for the UC-M and UC-F. *p*-values were calculated by using a Kruskal-Wallis non-parametric test, as described in the Methods: *P < 0.05, **P < 0.01, ***P < 0.001.

**Additional file 4.** Tables describing clones used and replicates performed, antibodies, cell counts, and data values. (.xlsx). **(A)** Indication of which clonal line was used for each biological replicate experiment and the number of biological replicates that were performed to procure the data for each figure panel. **(B)** Antibodies used for immunocytochemistry and immunoblotting, with the dilutions, suppliers, and host species indicated. **(C)** Number of Ki67-expressing NPCs and total number of DAPI-stained nuclei quantified to define the Ki67-expressing fraction in Fig. 2F. For Fig. 3D, total number of neurons used for quantitation of neurite length, based upon number of DAPI-expressing nuclei. Additional sheets provide data values for each figure panel involving <6 replicate experiments are also provided here or as indicated.

**Additional file 5. **Differentially expressed genes. (.xlsx). DEGs were obtained by pairwise comparisons of normalized RNA-seq expression data from the four models, including calculation of the log2 fold change, FDR corrected *p*-values (padj), and average RPKM values across the sample types analyzed. See Methods for further information. (*n* = 4).

**Additional file 6. **Ingenuity Pathways Analysis (IPA) of DEGs specific to the AP by comparison with the UM. (.xlsx). Significantly enriched: **(A)** Pathway and **(B)** Disease-related GO terms are shown, with the *p*-value and the DEGs from which each term was derived. **(C)** The top 1500 DEGs from the AP vs. UM comparison (based upon fdr/adj *p*-value) were input into the Percayai suite's CompBio pathway analysis tool to obtain networks associated with these DEGs. These networks were filtered with a concept filter utilizing the search terms "Wnt AND neuronal migration" to identify a network of interconnected themes (shown); concepts and underlying genes (entities) within each theme in the network above are shown. **(D)** Genes (entities) present in the Wnt AND Neuron Migration/Cadherin AND neural/interneuron themes are shown. These include two genes (*GJA1/TGFBR2*) that overlapped between Wnt-related and neuronal migration-related DEGs also called by Ingenuity Pathway Analysis. *n* = 4; data values are in Additional file 5.

**Additional file 7. **Differentially expressed genes in the AP, by comparison with all three other models. (.jpg). **(A)** Venn diagram shows numbers of differentially expressed genes (DEGs) obtained from pairwise comparisons of the AP versus (vs.) UM, AP vs. UC-M, and AP vs. UC-F models. AP-specific DEGs, based upon comparisons to at least two of the other datasets, are shaded in blue. These AP-specific DEGs were further analyzed by: **(B)** Hierarchical clustering analysis, visualizing comparisons with the other three sample types, and by (C-H) Ingenuity Pathway Analysis (IPA), which identified **(C)** enriched pathways and **(D)** disease-related GO terms. In C-D, the number of DEGs enriched for each term present is represented on the x-axis, with red and blue colors indicating up- and down-regulated genes, respectively. *p*-values for each enriched GO term are indicated. (E-F) IPA disease terms enriched in these AP-specific DEGs include gene networks associated with **(E)** Cellular movement and **(F)** Nervous system development and function. The numbers of up-and down-regulated genes present in the networks are indicated. Within each network, red and green symbols indicate up- and down-regulated genes respectively, while color intensity indicates the relative degree of differential expression. **(G)** Interaction network of differentially expressed genes with known significance in neurodevelopmental disorders (NDDs). In this network, the nodes are each of the genes, the size of the node corresponds to the size of the differential expression, and the color indicates the direction of the differential expression change (red = negative, blue = positive). *n* = 4; data values are in Additional file 5.

**Additional file 8. **Ingenuity Pathways Analysis (IPA) of DEGs specific to the AP, by comparison with two or more of the other (UM, UC-M, and/or UC-F) models. (.xlsx). Significantly enriched: **(A)** Pathway and **(B)** Disease-related GO terms are shown, with the *p*-value and the DEGs from which each term was derived. **(C-G)** All genes with differential expression between the AP and the three other models (gene list in C: comparison to UM, UC-M, UC-F) were compared with genes (D-E) that were significant in a transcription-wide association study (TWAS) of autism or that (F-G) had genome-wide significance for *de novo* protein-coding variants associated with neurodevelopmental disorders, as described in the results text. *n* = 4; data values are in Additional file 5.

**Additional file 9. **Differentially expressed genes in the UM, by comparison with the UC-M and UC-F control models. (.jpg). **(A)** Venn diagram shows numbers of differentially expressed genes (DEGs) obtained from pairwise comparisons between the UM vs. the UC-M or UC-F models. UM-specific DEGs are shown in blue. (B-F) These UM-specific DEGs were further analyzed by **(B)** Hierarchical clustering analysis, with comparisons to all three other models shown, and **(C)** using Ingenuity Pathway Analysis (IPA), which identified UM-enriched (C) pathways and **(D)** disease-related GO terms. In C-D, the number of DEGs enriched for each term present is represented on the x-axis, with red and blue colors indicating up- and down-regulated genes, respectively. *p*-values for each enriched GO term are indicated. (E-F) IPA disease terms enriched in these AP-specific DEGs include gene networks associated with **(E)** Nervous system development and function and **(F)** Behavior. The numbers of up-and down-regulated genes present in the networks are indicated. Within each network, red and green symbols indicate up- and down-regulated genes respectively, while color intensity indicates the relative degree of differential expression. *n* = 4; data values are in Additional file 5.

**Additional file 10. **Ingenuity Pathways Analysis (IPA) of DEGs specific to the UM, by comparison with the UC-M and UC-F models. (.xlsx). Significantly enriched: **(A)** Pathway and **(B)** Disease-related GO terms are shown, with the *p*-value and the DEGs from which each term was derived. *n* = 4; data values are in Additional file 5.

**Additional file 11. **AP-specific differential gene expression defined by RNA-seq analysis of differentiated cortical neuroids was validated by RT-qPCR and variancePartition analysis. (.jpg). Genes defined as differentially expressed in the AP, by comparison with the UM, by RNA-seq analysis were selected from top AP-enriched gene networks, including axon guidance molecules, integrins, ion channels, and transcription factors, and were validated by RT-qPCR. **(A)** RPKM values for these DEGs were obtained using RNA-seq analysis (*n* = 4; data values are in Additional file 5). **(B)** These were compared with relative gene expression in these models, as defined by RT-qPCR (*n* = 3; data values are in Additional file 4). RT-qPCR analysis was performed using samples obtained from three independent biological replicate experiments (*n* = 3) and was performed using a second set of clonal lines derived from the AP and UM that was different than the AP and UM clonal lines used for the RNA-seq analysis. *P* values *P < 0.05, **P < 0.01, ***P < 0.001 were determined by an unpaired t-test. (C) variancePartition analysis indicates the percent variance that is attributable to individuals from whom the samples were procured, and the study subjects’ age and sex.

**Additional file 12.** Schematic for maturation of cExNs and cINs. (.jpg). **(A)** Differentiation scheme used to obtain neurons for electrophysiology. cExNPCs and cINPCs, labelled respectively with Synapsin (Syn)-GFP and Syn–RFP, were differentiated in co-culture as cortical neuroids for 15 days, and then further matured by replating on a rat cortical astrocyte feeder layer. **(B)** MAP2 staining of dissociated cortical neuroids demonstrated that the UM-derived neurons had increased soma size, as quantified in Fig. 7A. (Scale bar = 75 μM). **(C)** Spontaneous fast excitatory (*) and slower inhibitory (^) postsynaptic currents recorded at -80 mV in a UM-derived cIN. The EPSC is replotted on a faster time base to the right.

**Additional file 13. **Comparison of electrophysiological properties of neurons derived *in vitro* from the UC-M, AP, and UM iPSC models. (.xlsx). Data is shown for **(A)** all cells (cExNs and cINs), **(B)** cExNs, and **(C)** cINs. (A) Data columns for cells of each genotype show the median, first, and third quartiles, and count (# of cells) for each parameter for all cells recorded, by combining data from cExNs and cINs for each genotype. Columns to the right show neuronal cell type-specific data, by combining cExN data or cIN data for all three genotypes. Light red shading denotes Significant Difference by 2-WAY ANOVA on ranks with post hoc comparison by Student-Newman-Keuls method (*p* < 0.05) with genotype (UC-M : AP : UM) and neuronal cell type (cExN : cIN) as the two factors. (B-C) Values for (B) cExNs or (C) cINs of each genotype are shown. Tan shading denotes Significant Difference by 1-WAY ANOVA on ranks with post hoc comparison by Student-Newman-Keuls method (*p* < 0.05), with darker tan indicating that one of the genotypes was different from the other two and lighter tan indicating that two of the three genotypes were different from each other. Capacitance and input resistance were determined from 10 mV voltage steps from -80 mV. First spike threshold, amplitude and half-width were determined for the first action potential recorded at threshold. Maximum first frequency and maximum average firing frequency were determined for 800 msec depolarizing steps that elicited the maximum number of spikes under current clamp. Peak inward sodium current, steady-state outward potassium currents and currents evoked by ACh and choline were recorded under whole-cell voltage clamp. Values are provided.

**Additional file 14.** Examples of genes in selected gene ontology terms described in text. (.pdf). Genes in selected classes are shown, with their classes, associated network and pathways, and physiological roles, and supporting references (cited in main text).

## Data Availability

All data generated and analyzed during this study are included in this published article, its additional information files, or in the publically available Gene Expression Omnibus (GEO) repository (Series GSE143908) [88]. Data values are provided in Additional files 4, 5, or 13, as indicated. iPSC lines used in this study are available from the investigator by material transfer agreement with Washington University.

## Abbreviations

ASD: Autism spectrum disorder
iPSCs: Induced pluripotent stem cells
CNV: Copy number variant
CHRNA7: α-7 nicotinic acetylcholine receptor subunit
ID: Intellectual disability
ADHD: Attention deficit and hyperactivity disorder
ER: Endoplasmic reticulum
GABA: Gamma-aminobutyric acid
RYR: Ryanodine receptor
UPR: Unfolded protein response
cExN: Cortical excitatory neuron
cIN: Cortical inhibitory neuron
NPC: Neural progenitor cell
UM: Unaffected mother
AP: Affected proband
UC-M: Unrelated control-male
UC-F: Unrelated control-female
GTAC: Genome Technology Access Center
ICC: Immunocytochemistry
RT-qPCR: Reverse transcription and quantitative PCR
EB: Embryoid body
DEG: Differentially expressed gene
IPA: Ingenuity Pathway Analysis
PI: Propidium iodide
PCA: Principal Component Analysis
GO: Gene ontology
