# Supplementary figures and images for: Altered neuronal physiology, development, and function associated with a common chromosome 15 duplication involving CHRNA7
Source: BMC Biol. 2021 Jul 28;19:147. doi: 10.1186/s12915-021-01080-7 (PMC8317352; doi:10.1186/s12915-021-01080-7)

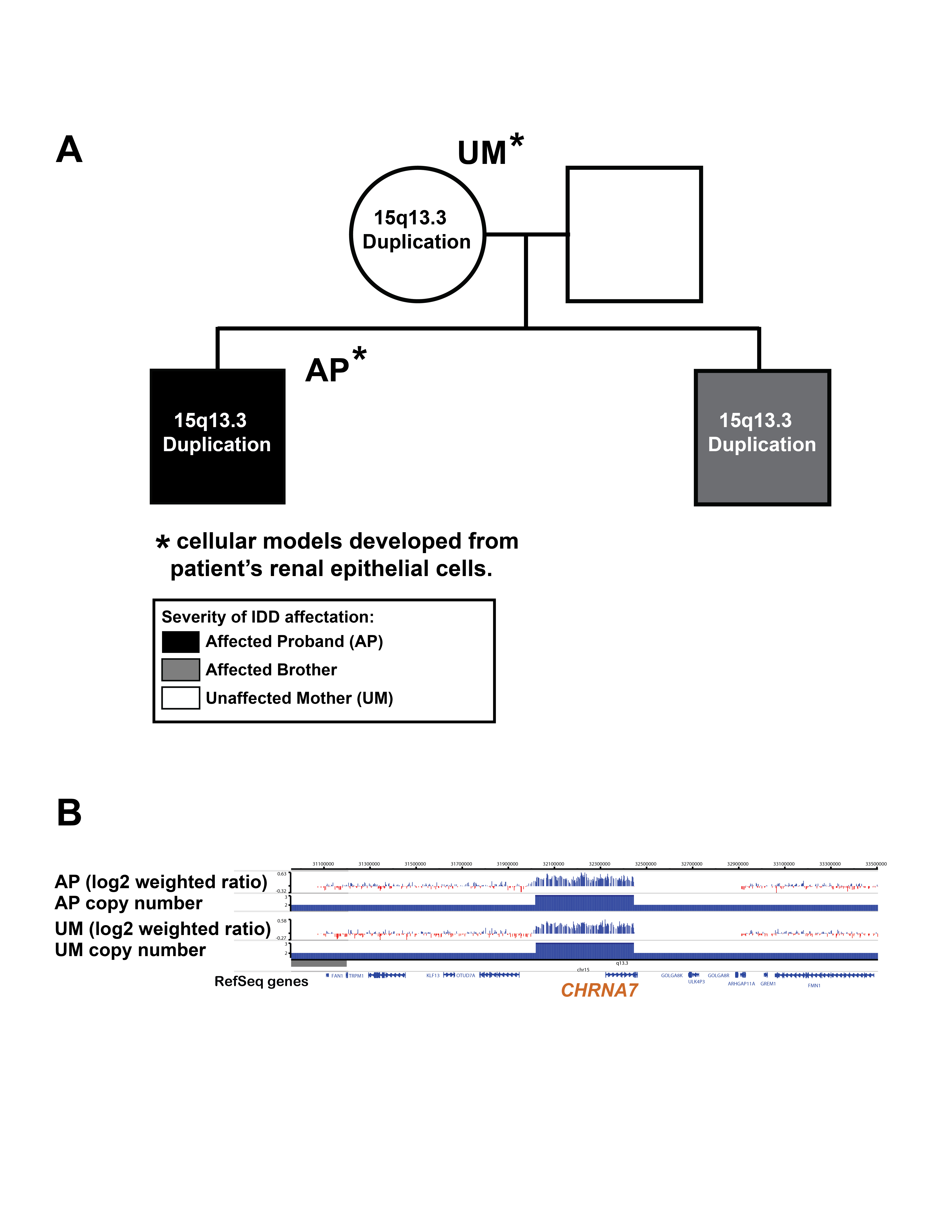

Supplement: Supplementary file 1 — Additional file 1. Characteristics of subjects in pedigree modeled here. (.jpg). (A) The study samples were derived from a pedigree with 15q13.3 duplication, with differential clinical affectation indicated by shading of subjects. The affected proband (AP) is represented in black, his affected brother, shown in gray, exhibits subtle autistic traits and has more volatile emotional dysregulation than the AP, while the unaffected mother (UM) and father are shown in white. Renal epithelial cells from the family members indicated (*) were used to derive iPSC models. (B) CNV array data for the AP and UM shows the signal intensity (log2 weighted ratio) and predicted copy number across the duplicated region of 15q13.3. The region lacking signal was not covered by the CNV array used for this analysis. [file 12915_2021_1080_MOESM1_ESM.jpg]

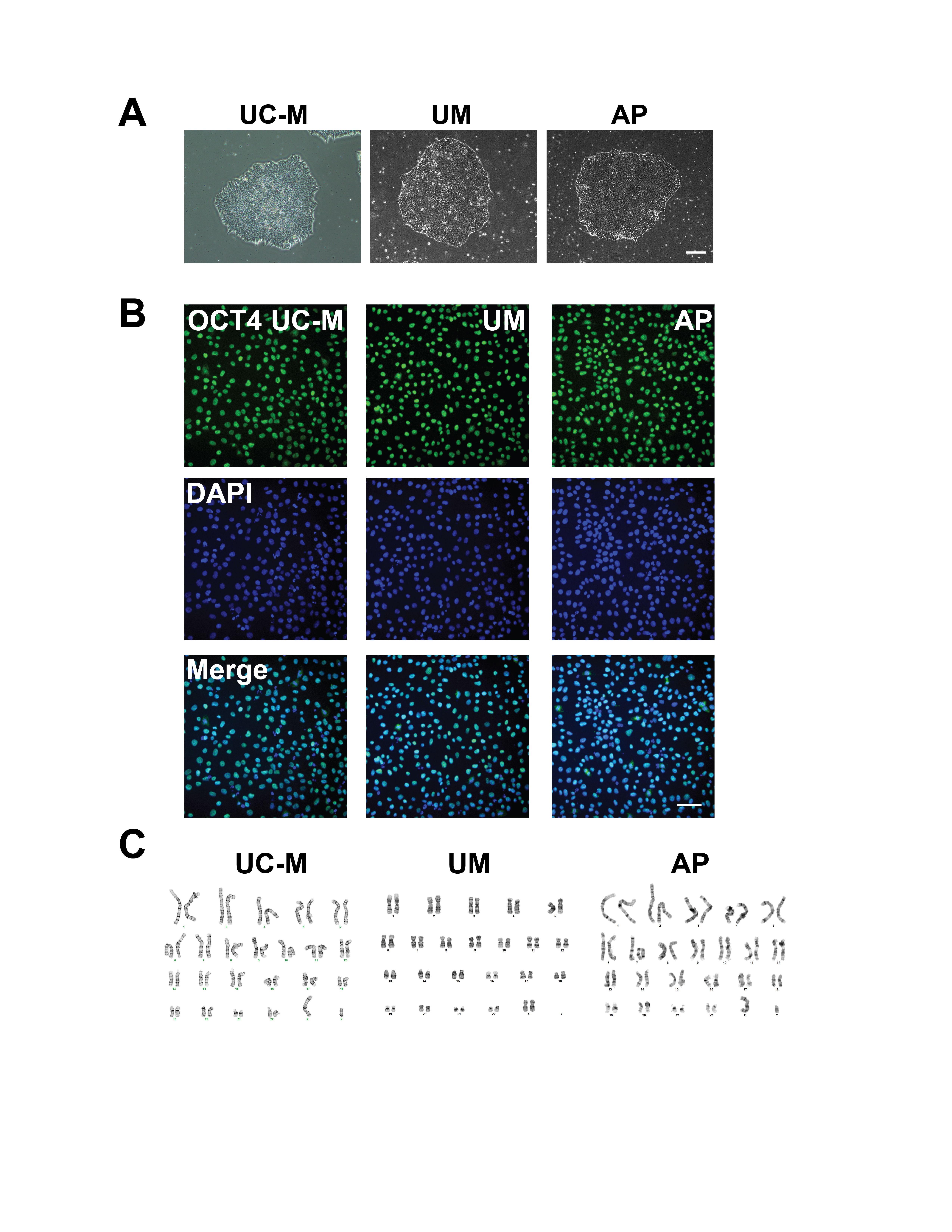

Supplement: Supplementary file 2 — Additional file 2. Characterization of iPSC models. (.jpg). Renal epithelial cell-derived iPSC lines from the UC-M, UM, and AP subjects (A) exhibit normal human stem cell colony morphology in bright field images (scale bar = 250 μm), (B) express the pluripotency marker OCT4/POU5F1 (scale bar = 150 μm), and (C) have a normal karyotype. [file 12915_2021_1080_MOESM2_ESM.jpg]

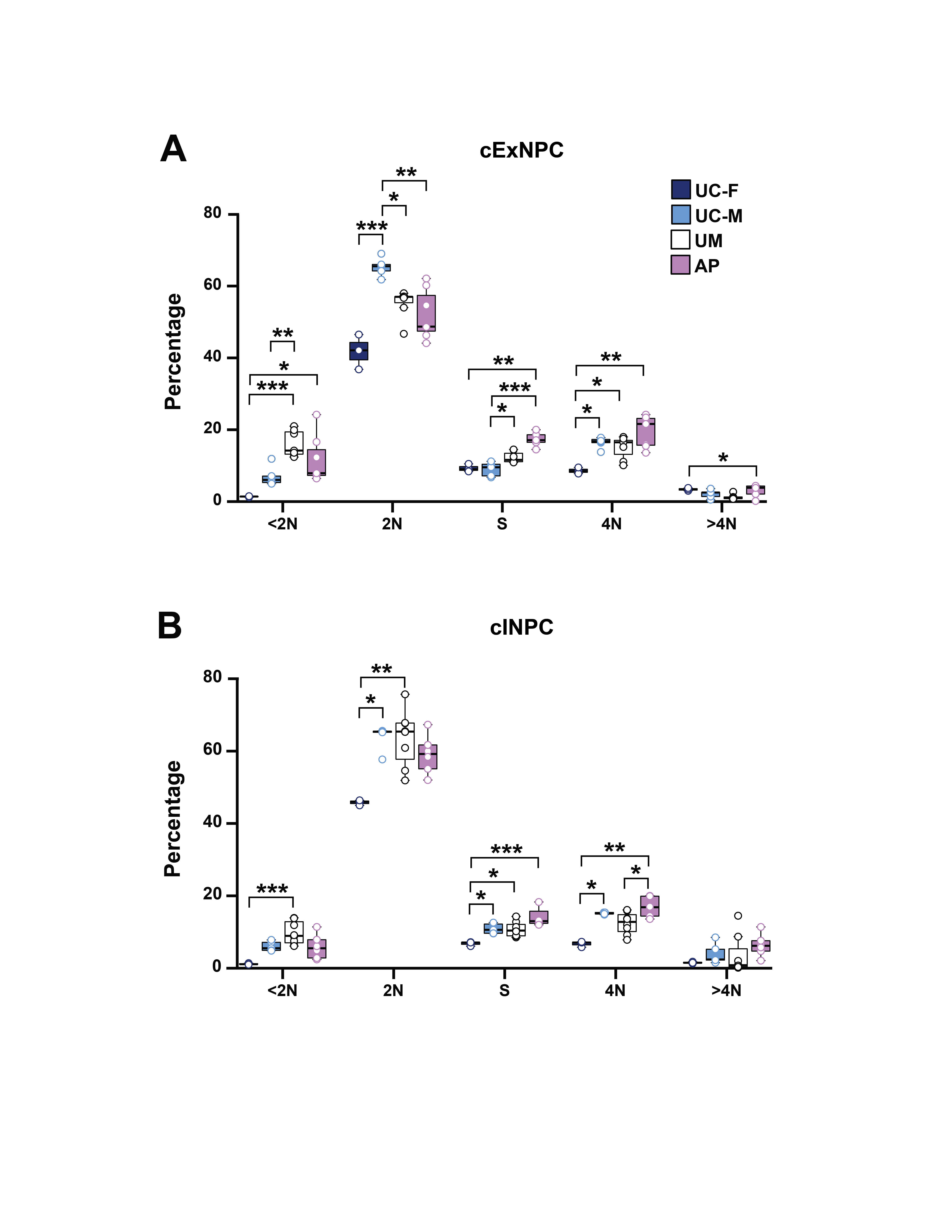

Supplement: Supplementary file 3 — Additional file 3 Cell cycle analysis of neural progenitor cells (NPCs). (.jpg). (A) cExNPCs and (B) cINPCs were stained with propidium iodide for DNA content and analyzed by FACS. Percentages of cells in each phase of the cell cycle were quantified for each model. Values shown are from seven independent biological replicate experiments (n = 7), using two clonal lines for the UM and AP, and one clonal line for the UC-M and UC-F. p-values were calculated by using a Kruskal-Wallis non-parametric test, as described in the Methods: *P < 0.05, **P < 0.01, ***P < 0.001. [file 12915_2021_1080_MOESM3_ESM.jpg]

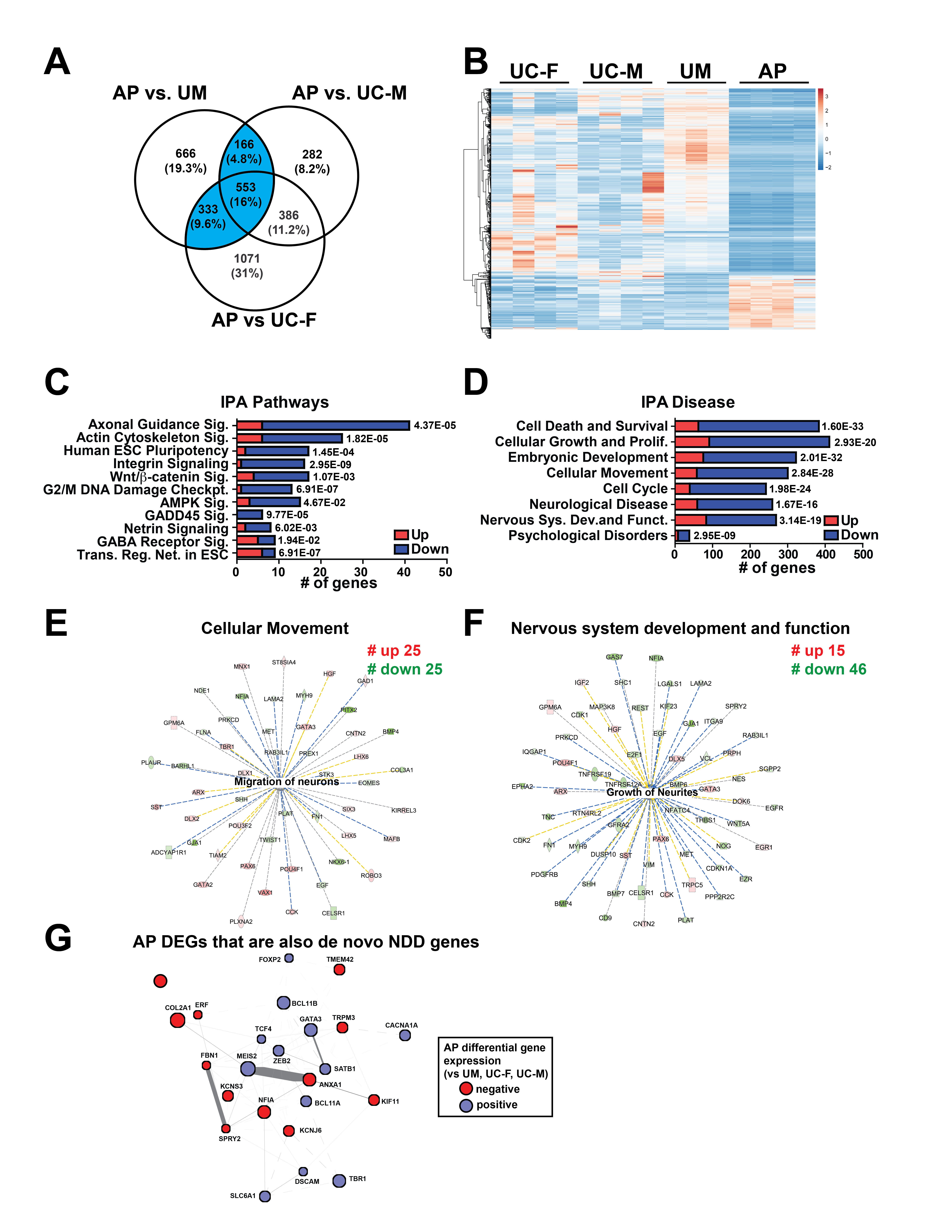

Supplement: Supplementary file 7 — Additional file 7. Differentially expressed genes in the AP, by comparison with all three other models. (.jpg). (A) Venn diagram shows numbers of differentially expressed genes (DEGs) obtained from pairwise comparisons of the AP versus (vs.) UM, AP vs. UC-M, and AP vs. UC-F models. AP-specific DEGs, based upon comparisons to at least two of the other datasets, are shaded in blue. These AP-specific DEGs were further analyzed by: (B) Hierarchical clustering analysis, visualizing comparisons with the other three sample types, and by (C-H) Ingenuity Pathway Analysis (IPA), which identified (C) enriched pathways and (D) disease-related GO terms. In C-D, the number of DEGs enriched for each term present is represented on the x-axis, with red and blue colors indicating up- and down-regulated genes, respectively. p-values for each enriched GO term are indicated. (E-F) IPA disease terms enriched in these AP-specific DEGs include gene networks associated with (E) Cellular movement and (F) Nervous system development and function. The numbers of up-and down-regulated genes present in the networks are indicated. Within each network, red and green symbols indicate up- and down-regulated genes respectively, while color intensity indicates the relative degree of differential expression. (G) Interaction network of differentially expressed genes with known significance in neurodevelopmental disorders (NDDs). In this network, the nodes are each of the genes, the size of the node corresponds to the size of the differential expression, and the color indicates the direction of the differential expression change (red = negative, blue = positive). n = 4; data values are in Additional file 5. [file 12915_2021_1080_MOESM7_ESM.jpg]

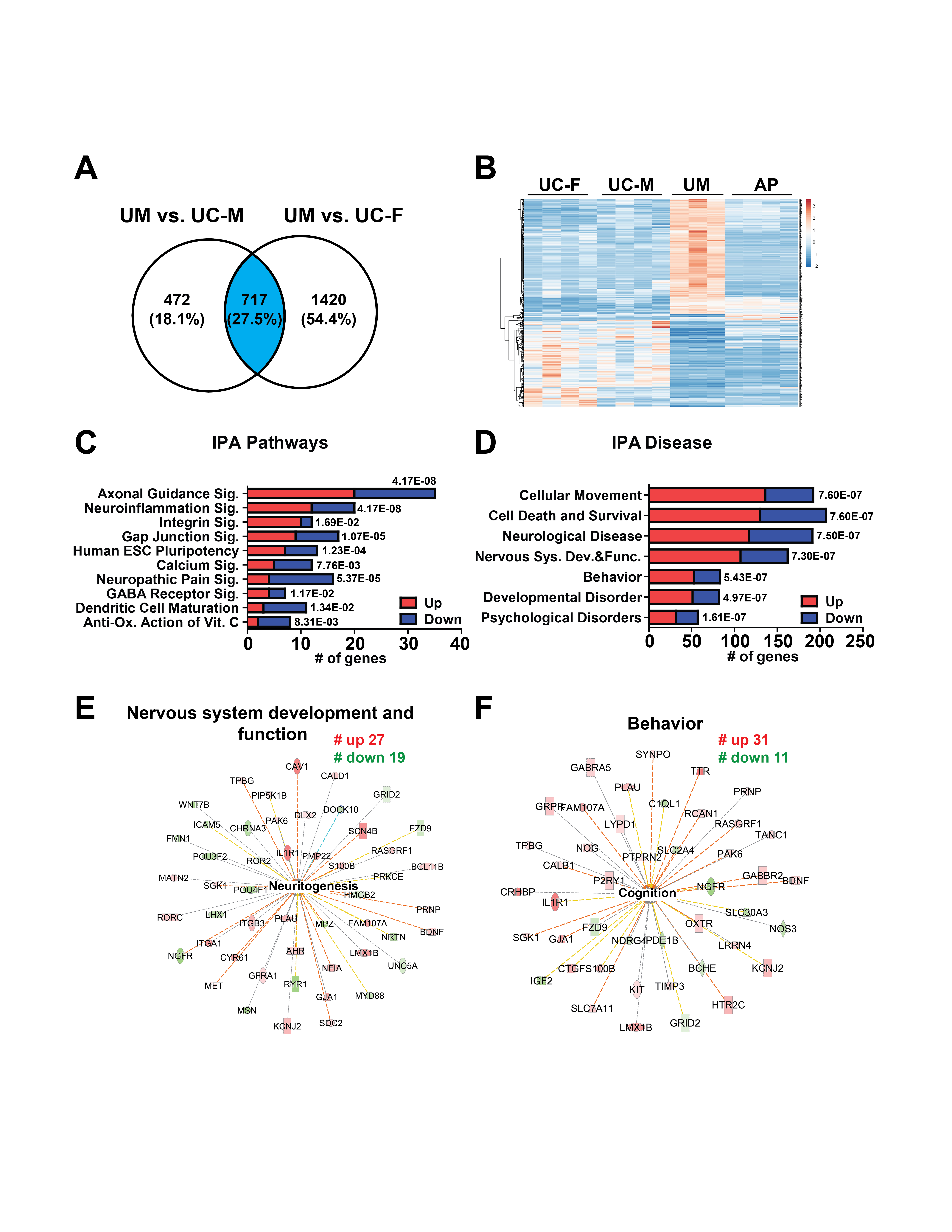

Supplement: Supplementary file 9 — Additional file 9. Differentially expressed genes in the UM, by comparison with the UC-M and UC-F control models. (.jpg). (A) Venn diagram shows numbers of differentially expressed genes (DEGs) obtained from pairwise comparisons between the UM vs. the UC-M or UC-F models. UM-specific DEGs are shown in blue. (B-F) These UM-specific DEGs were further analyzed by (B) Hierarchical clustering analysis, with comparisons to all three other models shown, and (C) using Ingenuity Pathway Analysis (IPA), which identified UM-enriched (C) pathways and (D) disease-related GO terms. In C-D, the number of DEGs enriched for each term present is represented on the x-axis, with red and blue colors indicating up- and down-regulated genes, respectively. p-values for each enriched GO term are indicated. (E-F) IPA disease terms enriched in these AP-specific DEGs include gene networks associated with (E) Nervous system development and function and (F) Behavior. The numbers of up-and down-regulated genes present in the networks are indicated. Within each network, red and green symbols indicate up- and down-regulated genes respectively, while color intensity indicates the relative degree of differential expression. n = 4; data values are in Additional file 5. [file 12915_2021_1080_MOESM9_ESM.jpg]

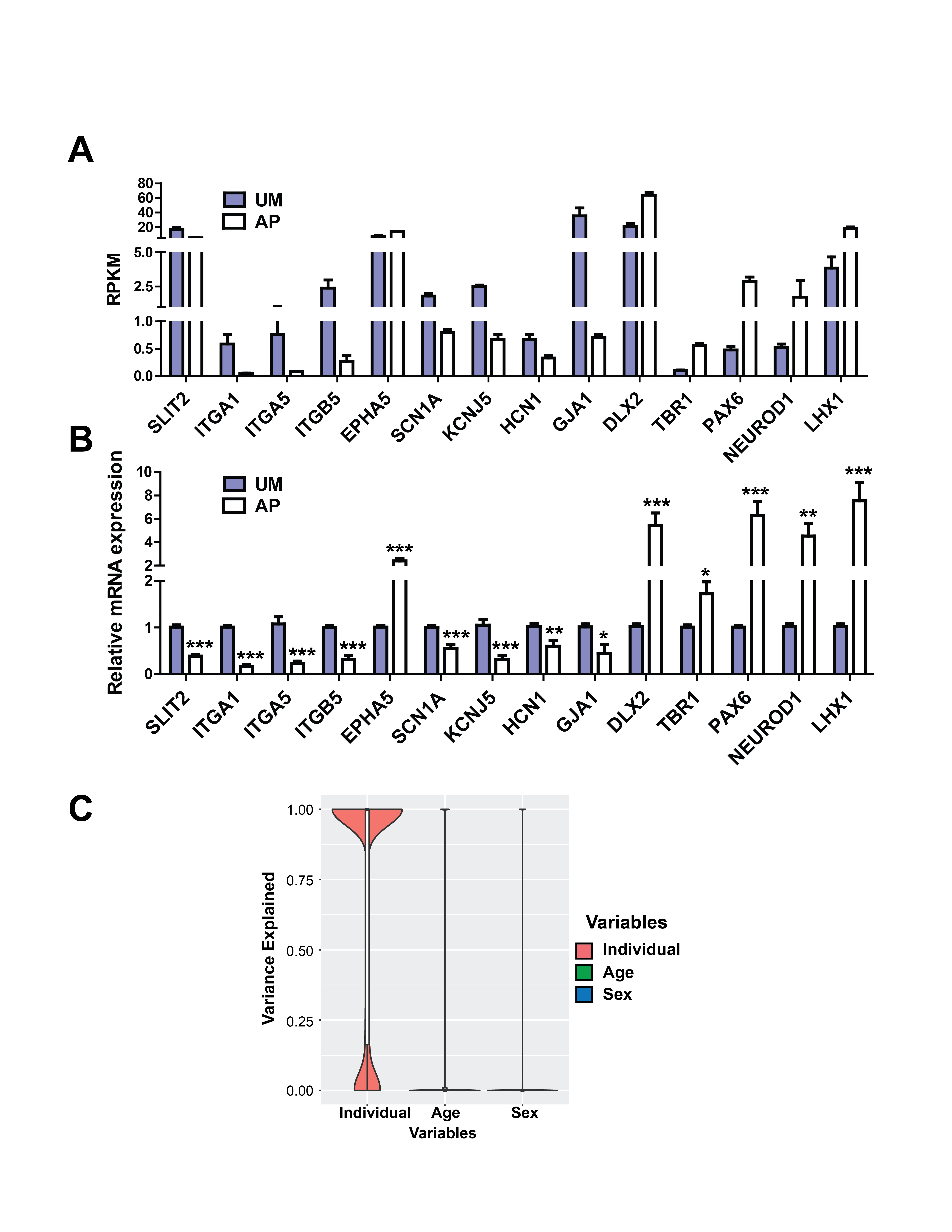

Supplement: Supplementary file 11 — Additional file 11. AP-specific differential gene expression defined by RNA-seq analysis of differentiated cortical neuroids was validated by RT-qPCR and variancePartition analysis. (.jpg). Genes defined as differentially expressed in the AP, by comparison with the UM, by RNA-seq analysis were selected from top AP-enriched gene networks, including axon guidance molecules, integrins, ion channels, and transcription factors, and were validated by RT-qPCR. (A) RPKM values for these DEGs were obtained using RNA-seq analysis (n = 4; data values are in Additional file 5). (B) These were compared with relative gene expression in these models, as defined by RT-qPCR (n = 3; data values are in Additional file 4). RT-qPCR analysis was performed using samples obtained from three independent biological replicate experiments (n = 3) and was performed using a second set of clonal lines derived from the AP and UM that was different than the AP and UM clonal lines used for the RNA-seq analysis. P values *P < 0.05, **P < 0.01, ***P < 0.001 were determined by an unpaired t-test. (C) variancePartition analysis indicates the percent variance that is attributable to individuals from whom the samples were procured, and the study subjects’ age and sex. [file 12915_2021_1080_MOESM11_ESM.jpg]

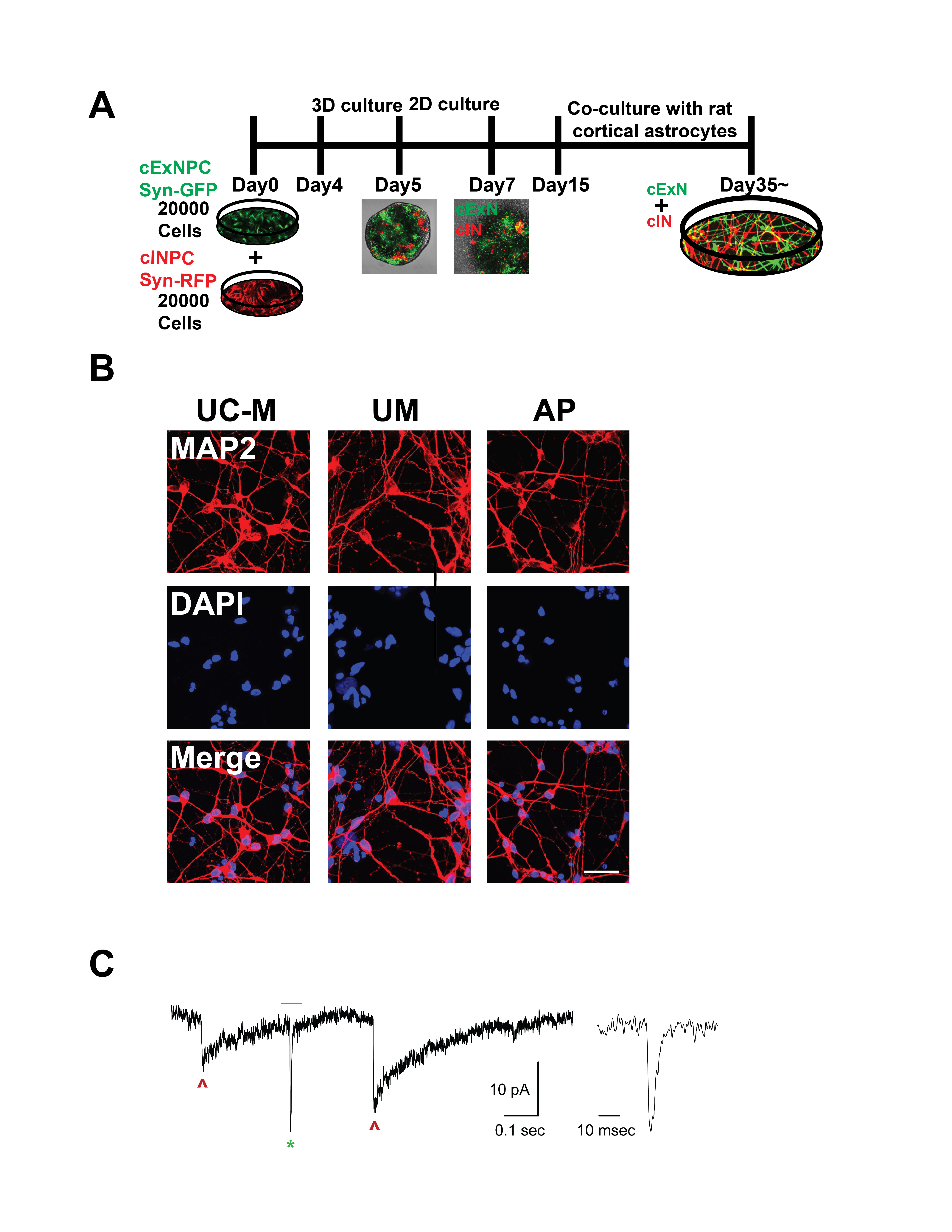

Supplement: Supplementary file 12 — Additional file 12. Schematic for maturation of cExNs and cINs. (.jpg). (A) Differentiation scheme used to obtain neurons for electrophysiology. cExNPCs and cINPCs, labelled respectively with Synapsin (Syn)-GFP and Syn–RFP, were differentiated in co-culture as cortical neuroids for 15 days, and then further matured by replating on a rat cortical astrocyte feeder layer. (B) MAP2 staining of dissociated cortical neuroids demonstrated that the UM-derived neurons had increased soma size, as quantified in Fig. 7A. (Scale bar = 75 μM). (C) Spontaneous fast excitatory (*) and slower inhibitory (^) postsynaptic currents recorded at -80 mV in a UM-derived cIN. The EPSC is replotted on a faster time base to the right. [file 12915_2021_1080_MOESM12_ESM.jpg]
