# Supplementary material for: Altered neuronal physiology, development, and function associated with a common chromosome 15 duplication involving CHRNA7
Source: BMC Biol. 2021 Jul 28;19:147. doi: 10.1186/s12915-021-01080-7 (PMC8317352; doi:10.1186/s12915-021-01080-7)
Supplement: Supplementary file 14 — Additional file 14. Examples of genes in selected gene ontology terms described in text. (.pdf). Genes in selected classes are shown, with their classes, associated network and pathways, and physiological roles, and supporting references (cited in main text). [file 12915_2021_1080_MOESM14_ESM.pdf]

Additional file 14. Examples of genes in selected gene ontology terms described in text

Gene Symbols	Class	Network/Pathways Identified	Physiological role	References
SLIT2, EPHA8, EPHA5, EPHB4, ROBO3, SLIT3, GJA1 and ROBO2	Axon guidance molecules	Axon guidance signaling	Axonal growth	(Jellinger et al., 1988; McFadden and Minshew, 2013; Wang et al., 2018)
ITGA1, ITGA2, ITGA3, ITGA5, ITGA9, ITGB3, ITGB4, and ITGB5	Cell adhesion molecules	Integrin Signaling	Synaptic development	(Carter et al., 2011; Lilja and Ivaska, 2018)
LMX1A, LMX1B, FOXB1, ARX, PAX6, DLX5, NEUROD1, and LHX1	Transcription factors	Behavior-Cognition, Psychological disorders,	Nervous system development and neuritogenesis	(Thanseem et al., 2011)
KCNJ2, KCNJ5, SCN1A, SCN4B, and HCN1	Channels	Nervous system development and function	Neuronal development	(Guglielmi et al., 2015; Thanseem et al., 2011)
TBR1, CNTNAP2, DLX2	Transcription factors and neurexin molecule	Neuritogenesis and migration of neurons	Neuronal migration	(Reiner et al., 2016); Blueprint Genetics
WNT8B, WNT10B, LEF1, WNT11, FZD2, FZD5, FRZB, FZD8, SFRP2, WNT7A, TCF4	WNT molecules	WNT signaling	Neuronal development and neuronal migration	(Bae and Hong, 2018; Kalkman, 2012; Kwan et al., 2016)
ARX, COL4A1, COL4A2, EMX2, FLNA, LAMA2, NDE1, OCLN, SRPX2, and WDR62	Transcription factors and others	Migration of neurons	Neuronal migration	Blueprint Genetics
